# Enhancing economic multifunctionality without compromising multidiversity and ecosystem multifunctionality via forest enrichment

**DOI:** 10.1126/sciadv.adp6566

**Published:** 2024-10-23

**Authors:** Larissa Regina Topanotti, Jasper M. Fuchs, Matthias Albert, Jan Schick, Alice Penanhoat, Jing-Zhong Lu, Carmen Alicia Rivera Pérez, Estela Covre Foltran, Scott Appleby, Benjamin Wildermuth, Thalea Stuckenberg, Likulunga Emmanuel Likulunga, Jonas Glatthorn, Andreas Schuldt, Andrea Polle, Niko Balkenhol, Stefan Scheu, Christian Ammer, Carola Paul, Nathaly Guerrero-Ramírez

**Affiliations:** ^1^ Department of Forest Economics and Sustainable Land-Use Planning, University of Göttingen, Büsgenweg 1, 37077 Göttingen, Germany.; ^2^ Universidade Federal de Santa Catarina, Divisão de Atividades Agropecuárias, Campus Curitibanos, Rodovia Ulysses Gaboardi km 03, 89520-000 Curitibanos, Brazil.; ^3^ Forest Resources Management, Institute of Terrestrial Ecosystems, Department of Environmental Systems Science, ETH Zurich, Universitätsstrasse 16, 8092 Zurich, Switzerland.; ^4^ Department of Forest Growth, Northwest German Forest Research Institute, Grätzelstr. 2, 37079 Göttingen, Germany.; ^5^ Faculty of Forest Sciences and Forest Ecology, University of Göttingen, Büsgenweg 5, 37077 Göttingen, Germany.; ^6^ Department of Spatial Structures and Digitization of Forests, University of Göttingen, Büsgenweg 1, 37077 Göttingen, Germany.; ^7^ Department of Silviculture and Forest Ecology of Temperate Zones, University of Göttingen, Büsgenweg 1, 37077 Göttingen, Germany.; ^8^ J.F. Blumenbach Institute of Zoology and Anthropology, University of Göttingen, Untere Karspüle 2, 37073 Göttingen, Germany.; ^9^ Department of Forest Botany and Tree Physiology, University of Göttingen, Büsgenweg 2, 37077 Göttingen, Germany.; ^10^ Biodiversity and Evolution of Plants, Institute of Biology and Environmental Sciences, Carl von Ossietzky University of Oldenburg, Ammerländer Heerstr. 114-118, 26129 Oldenburg, Germany.; ^11^ French National Institute for Agriculture, Food and Environment (INRAE), 33140 Villenave-d’Ornon, Bordeaux, France.; ^12^ Department of Wildlife Sciences, University of Göttingen, Büsgenweg 3, 37077 Göttingen, Germany.; ^13^ Department of Forest Nature Conservation, University of Göttingen, Büsgenweg 3, 37077 Göttingen, Germany.; ^14^ Institute of Ecology and Evolution, University of Jena, Dornburger Str. 159, 07743 Jena, Germany.; ^15^ German Centre for Integrative Biodiversity Research (iDiv) Halle-Jena-Leipzig, Puschstr. 4, 04103 Leipzig, Germany.; ^16^ Biological Sciences Department, University of Zambia, Great East Road Campus, 32379 Lusaka, Zambia.; ^17^ Swiss Federal Research Institute WSL, Zürcherstrasse 111, 8903 Birmensdorf, Switzerland.; ^18^ Centre of Biodiversity and Sustainable Land Use, University of Göttingen, Büsgenweg 1, 37077 Göttingen, Germany.

## Abstract

Enriching tree species–poor and less productive forests by introducing economically valuable species is a strategy proposed for achieving multipurpose forest management. However, empirical evidence from managed and mature forests on the impact of this enrichment on ecological (multidiversity and ecosystem multifunctionality) and economic dimensions remains scarce, particularly when nonnative species are used. Here, we propose and test a framework that integrates economic multifunctionality, encompassing timber production–oriented goals and resistance against disturbances, with multidiversity and ecosystem multifunctionality in European beech forest stands enriched with conifers. Our results show that enriched beech forest stands (~80 years old) can provide high levels of economic multifunctionality without compromising multidiversity and ecosystem multifunctionality. In comparison to pure beech stands, enriched stands with Douglas-fir supported win-win-win situations for these three dimensions. Our findings contribute to the discussion of integrating biodiversity, ecosystem, and economic functions, providing empirical evidence for future forest management.

## Teaser

Enrichment of tree species–poor forests is a strategy to generate win-win-win situations in multipurpose forest management.

## INTRODUCTION

Forests are central in providing and maintaining biodiversity, ecosystem functioning, and economic contributions to society (*1*). However, increasing global demand for wood and fiber has often led to prioritizing wood production in forest management. According to the Global Forest Resource Assessment, 30% of the world’s forests are used and managed primarily for production-oriented objectives (*1*). In recent decades, this focus has often reduced tree species diversity to a few valuable timber species, with timber plantations of single, mostly nonnative species, making up 45% of planted forests (*1*). Given the relevance of timber plantations, there is a growing call for a multidimensional perspective focusing on multipurpose and resilient forests (*2*). To achieve this, designing forest management strategies that reconcile ecological and economic dimensions while considering different immediate and medium-term economic goals and the resistance against disturbances is needed.

Enriching less productive forests with economically favorable species has been considered a promising forest management strategy to fulfill different private and societal objectives (*3*, *4*). Enrichment is an interesting approach in forest management compared to alternatives relying on transformations to productive monocultures (*5*–*7*). Such a strategy may be viable in different parts of the world, particularly in management situations where native broadleaved species occur naturally in pure stands—due to the species’ high competitiveness (*8*). One example is the European beech forest, which covers large parts of Europe (*8*, *9*). In such cases, introducing highly productive coniferous species may enhance the economic functions of the forests. Further, enriching a tree species–poor forest increases tree diversity, which has been observed to positively affect the diversity of a wide range of associated taxa (*10*, *11*) [henceforth multidiversity (*12*)] and multiple ecosystem functions (*13*–*15*) [henceforth ecosystem multifunctionality (*16*)]. Yet, it remains unclear whether positive ecological effects may be generated by adding only one functionally different and commercially attractive species to tree species–poor forests and monocultures—a realistic scenario in managed forests and forest plantations worldwide (*3*, *17*–*19*).

The species in an enrichment system may be selected because of their high productivity, resistance to abiotic and biotic global change drivers, or both (*20*–*22*). For example, to guarantee high-performing forestry production systems, it is essential to identify which tree species composition reduces trade-offs among different management goals (*23*). However, the species’ desired characteristics may not be available in the regional species pools, particularly when considering tree species bottleneck for forest management due to end-of-century climate conditions (*20*). Therefore, creating mixed forests using nonnative species may become a vital adaptation strategy in forestry in the face of climate change (*20*, *24*). Despite the benefits associated with the cultivation of nonnative tree species, they can potentially become invasive (*21*) and detrimentally affect biodiversity and ecosystem functioning (*25*–*28*), with these effects likely depending on the resident time after introduction (*29*). Yet, most experimental studies assessing the impact of mixed forests on biodiversity and ecosystem functioning have excluded nonnative species or mature stands [e.g., Belluau *et al.* (*30*)]. Therefore, empirical evidence from mature forests is needed because economic benefits, mainly when nonnative species are used, should not come at the expense of multidiversity and ecosystem multifunctionality.

**Fig. 1.**
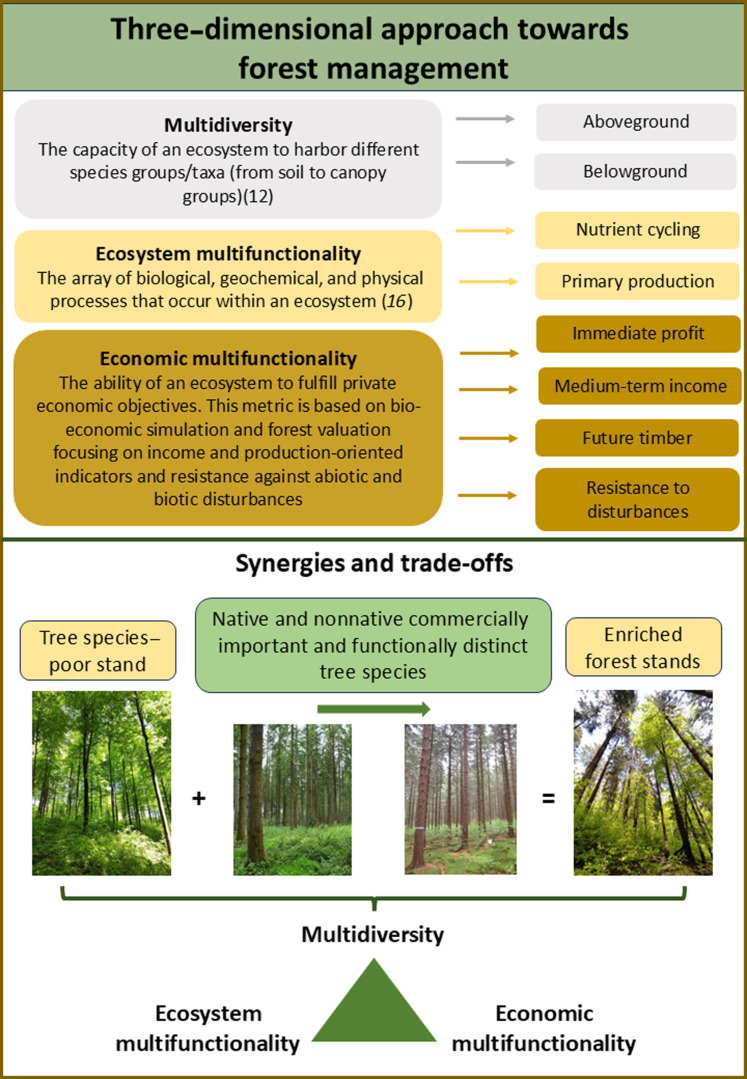
Conceptual figure illustrating our proposed framework for multipurpose forest management with the integration of economic multifunctionality. The figure includes the definition of multidiversity, ecosystem multifunctionality, and economic multifunctionality (upper part, boxes on the left) as well as the groups of taxa/functions (boxes on the right), which are reflected by indicators shown in tables S1 and S2. First, we propose an integrative index of economic multifunctionality. Second, we adopt an ecosystem perspective by integrating multidiversity, ecosystem multifunctionality, and economic multifunctionality in the context of multipurpose forest management. We aim to understand how the enrichment of tree species–poor forests with native and nonnative commercially valuable and functionally distinct tree species affects the provision levels of these three dimensions of forest management and their relationships. Photo credits: M. Spielmann, NW-FVA.

Relationships between tree diversity and ecosystem multifunctionality have been studied more intensively, pointing toward positive biodiversity-ecosystem multifunctionality relationships (*13*, *31*–*35*). In contrast, relationships between ecological and economic dimensions have often been limited to measuring yield (*36*), profit (*37*), biomass production [a weak indicator for income (*38*, *39*)], or long-term income (*40*, *41*) [but see Knoke *et al.* (*42*)]. However, the economic goals of a private beneficiary focusing on timber production and income comprise both immediate and long-term perspectives. Therefore, a holistic perspective is needed, which includes indicators considering multiple time frames (i.e., immediate and medium-term income) and risk-related measures (e.g., resistance against abiotic and biotic disturbances). Here, we integrated multiple economic goals as “economic multifunctionality” (Fig. 1). While economic multifunctionality focuses on production-oriented objectives, as we assume them to be relevant drivers of forest owners’ decisions (*43*–*45*), the concept has the potential to be extended to include further forest benefits (*16*, *46*). By proposing economic multifunctionality, we extended our current knowledge of the relationships between the provision of multidiversity, ecosystem multifunctionality, and economic benefits preferred by land managers (*16*, *46*) by explicitly considering temporal and risk-related measures from an economic perspective. We refer to multidiversity, ecosystem multifunctionality, and economic multifunctionality as the three dimensions of multipurpose forest management. This allows us to build a three-dimensional framework (Fig. 1), given that multipurpose forest management requires (i) a wide range of indicators from ecological (i.e., multidiversity and multifunctionality) and economic dimensions and (ii) understanding potential synergies and trade-off among them to subside stakeholder decisions.

Here, we empirically test our three-dimensional forest multifunctionality framework. Specifically, we assessed (i) the effects of enriching tree species–poor forests with a commercially important and functionally dissimilar tree species, either native or nonnative, on each of these dimensions; and (ii) the impacts of enrichment on the relationships between the three dimensions of multifunctional forest management. These relationships may be positive [synergies, i.e., a win-win situation involving mutual improvement of both characteristics (*47*)], negative [trade-offs/win-lose; losing one quality of something in return for gaining another (*23*, *48*, *49*)], or neutral. We hypothesized that (i) enrichment of tree species–poor forests with highly productive (coniferous) species enhances multidiversity, ecosystem multifunctionality, and economic multifunctionality because of positive tree diversity effects. In addition, we hypothesized that (ii) enriching tree species–poor forests with productive tree species reduces negative ecological impacts compared with forest management alternatives such as pure stands of productive species. This likely results in a win-win-win between ecological (i.e., ecosystem multifunctionality and multidiversity) and economic dimensions for enriched forests versus trade-offs expected for pure stands. While we expected an overall win-win between ecosystem multifunctionality and economic multifunctionality, as highly productive forest stands are not necessarily those with lower provision of ecosystem functions (*23*), we hypothesized (iii) trade-offs between economic and ecological dimensions in the presence of nonnative species.

We empirically tested this framework using enriched European beech forests as a model system. This forest type is relevant because beech (*Fagus sylvatica* L.; beech) is one of the most widespread broadleaved trees in Europe (*9*) naturally occurring in pure stands (*50*). Further, looking at economic multifunctionality in this forest type is crucial, given that forest owners’ income in Central Europe comes almost exclusively from timber (*43*, *51*, *52*). As in many regions worldwide, nonnative but productive species or species outside their natural range have been introduced over the last century, usually as pure stands (*17*, *53*). The planting of such species, in beech forests, was motivated by a need to increase the productive value of forests, given the lower productivity, less favorable wood properties, and prices of beech (*53*). Specifically, Norway spruce (*Picea abies* [L.] Karst.; spruce), native to mountainous regions of Europe (*51*, *54*), and the nonnative Douglas-fir (*Pseudotsuga menziesii* [Mirbel] Franco.) were introduced, with Douglas-fir considered as potentially better adapted to climate change (*55*) and having higher productivity than native alternatives (*22*, *51*, *56*, *57*).

We use a unique dataset from 40 plots distributed across eight locations in Germany representing tree species–poor forests (baseline, i.e., pure beech stands), two enrichment scenarios (i.e., beech-spruce and beech–Douglas-fir mixtures), and the introduced species in monodominant stands (i.e., pure spruce and pure Douglas-fir stands; henceforth forest types, fig. S1). Here, we focus on data from the stand scale (alpha-diversity), the unit where management decisions are planned and conducted. It is particularly relevant for small forest-holders (up to 20 hectares), which account, for example, for ~25% of the forest area in Germany (*51*). Multidiversity was quantified by an integrative index (*12*, *58*) representing species richness across seven forest biodiversity indicators from soil to canopy. We considered ecosystem multifunctionality as “the array of biological, geochemical, and physical processes that occur within an ecosystem” (*16*), including eight indicators as proxies for primary production, nutrient cycling–related drivers/processes, and tree recruitment (*16*, *23*, *31*). We define economic multifunctionality as the ability of an ecosystem to fulfill private economic objectives. Our six economic indicators focus on the management goals of a private beneficiary, represented by income and production-oriented indicators (*56*) and stand resistance against abiotic and biotic disturbances accounting for climate change (*40*, *59*, *60*) (tables S1 and S2). For these three integrative indexes, i.e., multidiversity, ecosystem multifunctionality, and economic multifunctionality, we calculated the effective number of taxa or functions, i.e., the actual weighted sum of the (normalized) taxa or performance level (*q* = 0) following the Hill-Chao approach (*61*). The approach allows accounting for correlations among the single taxon or functions considered.

## RESULTS

### Effects of enrichment on multidiversity, ecosystem multifunctionality, and economic multifunctionality

**Fig. 2.**
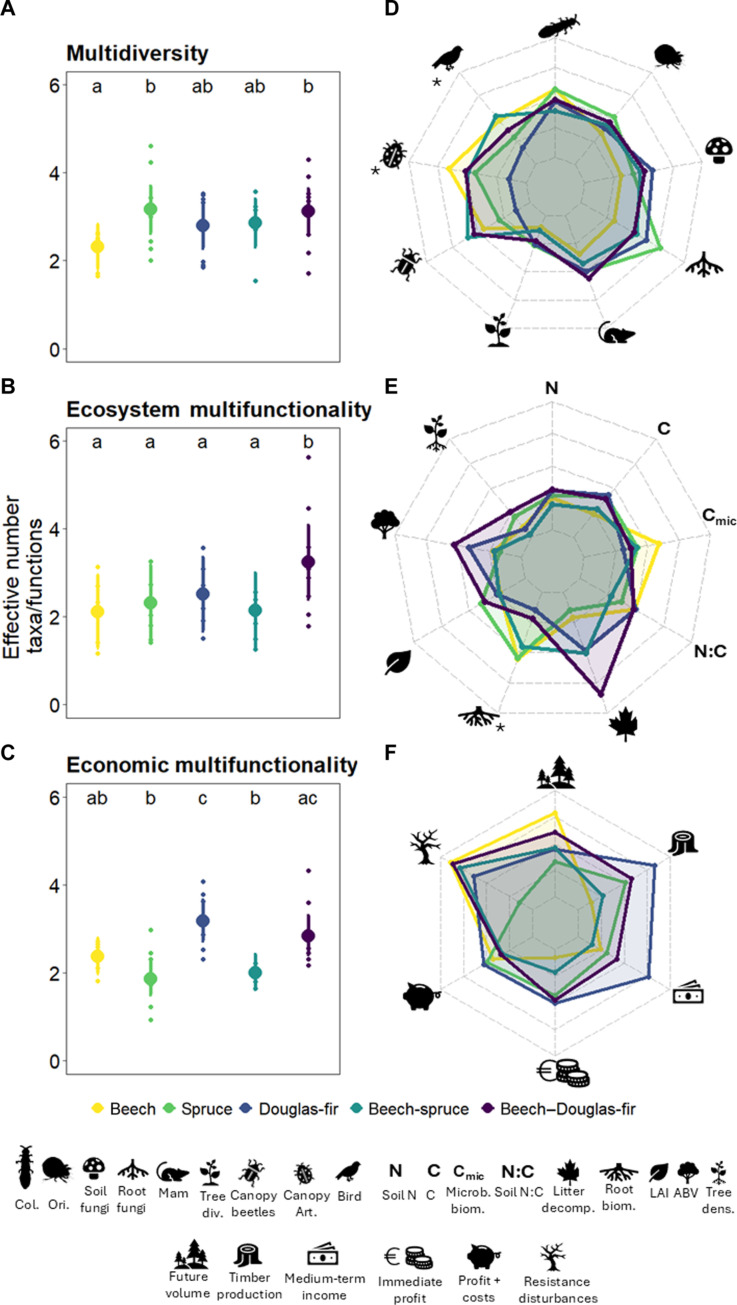
Effective number of taxa or functions for multidiversity, ecosystem multifunctionality, and economic multifunctionality (MF), across pure and enriched stands. The Hill-Chao approach represents the effective number of taxa or functions correcting for correlation between them (*61*). (**A**) to (**C**) show variation among forest types, which include beech, spruce, Douglas-fir, mixtures of beech and spruce, and mixtures of beech and Douglas-fir. On the basis of generalized linear mixed-effect models (see Materials and Methods), forest type influenced multidiversity, ecosystem multifunctionality, and economic multifunctionality (*P* < 0.05, table S3). Lowercase letters indicate significant differences between forest types based on pairwise Tukey’s significance test at 95% probability. We measured nine indicators of biodiversity (**D**) and ecosystem multifunctionality (**E**) and six indicators of economic multifunctionality (**F**). Indicators include Col = Collembola, Ori = Oribatida, Mam = small mammals, Tree div = tree diversity, canopy art = canopy arthropods, soil N = soil nitrogen stock, C = soil carbon stock, Microb biom = microbial biomass, litter decomp = litter decomposition, root biom = root biomass, LAI = leaf area index, ABV = aboveground tree biomass, and tree dens = tree density (see tables S1 and S2 for a detailed description of these indicators). Indicators followed by an asterisk (*) were not included in the calculations of the integrative measures due to data not being available for all plots (see Materials and Methods).

Forest type had significant effects on multidiversity (χ^2^ = 10.35, *P* = 0.03), ecosystem multifunctionality (χ^2^ = 26.4, *P* < 0.001), and economic multifunctionality (χ^2^ = 36.83, *P* < 0.001; table S3). For multidiversity, spruce and the beech–Douglas-fir mixture showed a higher effective number of taxa than pure beech (+36.0 and 34.5%, respectively; Fig. 2A and table S4). For ecosystem multifunctionality, the beech–Douglas-fir mixture showed the highest effective number of functions (e.g., by 52.4% higher compared with pure beech stands, Fig. 2B and table S4). Economic multifunctionality was higher in forest stands with Douglas-fir (in both the mixed and pure stand forest type), followed by beech and lower in stands with spruce (in both the mixed and pure stand forest type) (Fig. 2, C and F). This difference amounted to a 30 and 24% higher economic multifunctionality (measured as the effective number of economic functions) in pure Douglas-fir stands (3.17) and beech–Douglas-fir mixture (2.84), respectively, compared to spruce stands (1.86). This low spruce performance (in mixed and pure stands) and high performance of Douglas-fir and beech–Douglas-fir were robust when using a threshold approach instead of the Hill-Chao approach (fig. S2).

Looking at individual biodiversity indicators reveals that four of nine were influenced significantly by forest type (i.e., soil fungi, root fungi, canopy beetles, and canopy arthropods; *P* < 0.05, Fig. 2D, table S3, and fig. S3). On the one hand, the presence of conifers (in mixed and pure stands) resulted in higher species richness of soil and root fungi compared to pure beech plots. On the other hand, for biodiversity indicators associated with canopy biodiversity, pure Douglas-fir stands harbored lower species richness of canopy beetle and arthropod than other forest types (fig. S3). For most of the taxa, the highest proportion of species was shared among all forest types, ranging from 14% for canopy beetles to 50% for small mammals; the exception was tree diversity via natural regeneration, with only 12% of the species shared among forest types (fig. S4). The added proportion of species found only in a unique forest type was zero in the case of small mammals, 10% soil fungi, 20% Collembola (with unique species only found in monocultures), 23% Oribatida, 24% for birds (with zero unique species in monocultures of conifers), 31% root fungi, 41% canopy beetles, and 48% tree diversity, with the highest percentage of unique species in conifer pure stands and beech–Douglas-fir mixture) (fig. S4).

For the individual indicators of ecosystem functioning, forest type significantly affected six of the nine ecosystem functions [i.e., soil carbon (C) stock, microbial biomass, soil N:C ratio, litter decomposition, aboveground tree biomass, and fine root biomass; *P* < 0.05, Fig. 2E, table S3, and fig. S5]. Differences for soil C and microbial biomass were mainly associated with the lowest and highest values for pure beech stands compared with pure stands of spruce and Douglas-fir, respectively. For soil N:C ratio, root biomass, and aboveground biomass, significant differences were associated with the presence of Douglas-fir (in mixture and pure stands), with higher soil N:C ratio compared with beech-spruce mixture, lower root biomass (either mixture or pure stands) compared with other forest types, and highest aboveground biomass, particularly in mixtures. For litter decomposition, significant differences were associated with faster decomposition in mixtures compared with their respective pure stands, with the highest mass loss in beech–Douglas-fir mixture.

Of six economic functions, five were significantly influenced by forest type except for immediate profit reduced by establishment costs (*P* < 0.001, Fig. 2F, table S3, and fig. S6). The presence of Douglas-fir (either as a mixture or pure stands) drove high levels of future timber production, immediate profit, and medium-term income. In contrast, significantly higher resistance against abiotic and biotic disturbances was associated with pure beech stands and mixtures (with spruce and Douglas-fir) compared with pure spruce stands, and future wood volume was associated with higher values in pure beech stands and lower values in pure spruce stands. 

**Fig. 3.**
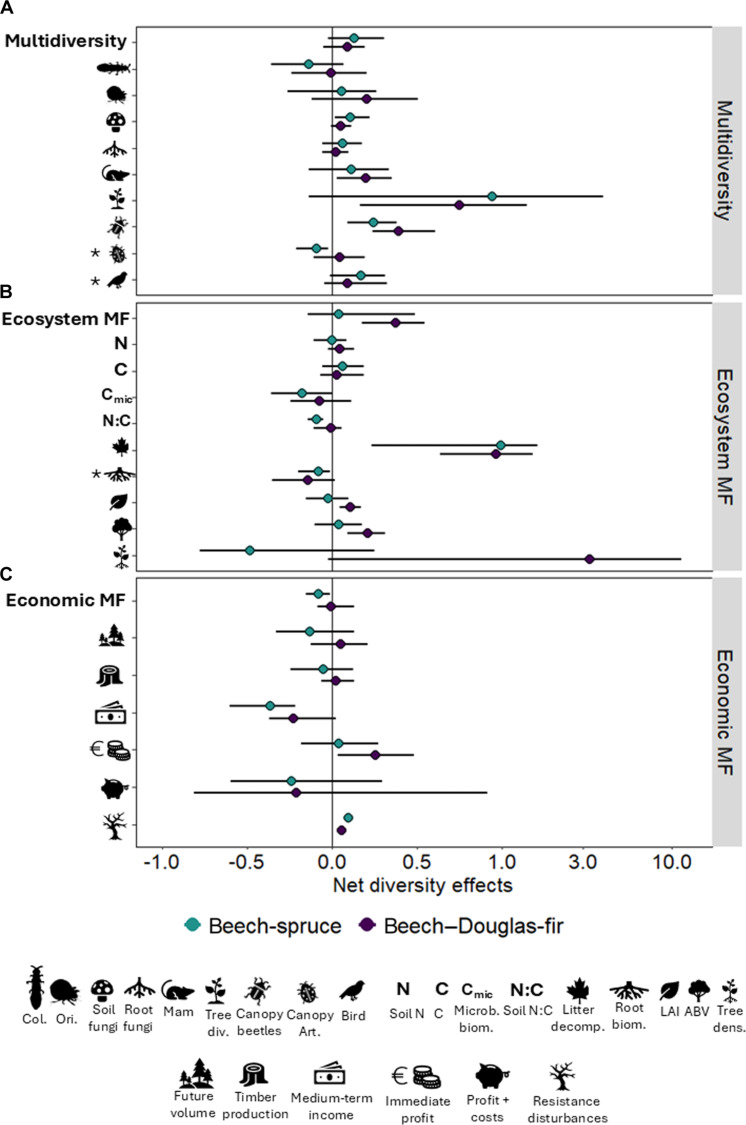
Net diversity effects calculated for multidiversity, ecosystem multifunctionality, and economic multifunctionality and their individual indicators [mean ± 95% confidence intervals (CIs)]. Net diversity effects were calculated using proportional deviance, i.e., comparing observed from mixtures and expected values based on pure stands for (**A**) multidiversity, (**B**) ecosystem multifunctionality and (**C**) economic multifunctionality. Net diversity effects were calculated for the two enrichment systems: beech-spruce and beech–Douglas-fir (see Materials and Methods). Nonadditive effects occur when the 95% CIs do not overlap with zero. Indicators include Col = Collembola, Ori = Oribatida, Mam = small mammals, Tree div = tree diversity, canopy art = canopy arthropods, soil N = soil nitrogen stock, C = soil carbon stock, Microb biom = microbial biomass, litter decomp = litter decomposition, root biom = root biomass, LAI = leaf area index, ABV = aboveground tree biomass, and tree dens = tree density (see tables S1 and S2 for a detailed description of these indicators). Indicators followed by an asterisk (*) were not included in the multidiversity or ecosystem multifunctionality calculations. The horizontal scale was log10 transformed for values higher than 1 to improve the visualization of the effects.

We found positive diversity effects, i.e., nonadditive effects when comparing observed values in mixtures and expected values based on pure stands, on ecosystem multifunctionality for beech–Douglas-fir mixture. For the individual indicators, consistent positive diversity effects were observed for canopy beetle richness, litter decomposition, and resistance to abiotic and biotic disturbances (tree survival probability). In addition, positive effects explicitly linked to Douglas-fir enrichment were found for small mammals, diversity of trees via natural regeneration, leaf area index (LAI), aboveground biomass, and immediate profit. Positive effects associated with spruce enrichment were observed for soil fungi. In contrast, nonadditive negative effects were observed for spruce enrichment on economic multifunctionality, canopy arthropods, soil N:C ratio, and medium-term income, i.e., annuity (Fig. 3, A to C).

### Trade-offs and synergies between multidiversity, ecosystem, and economic multifunctionality

**Fig. 4.**
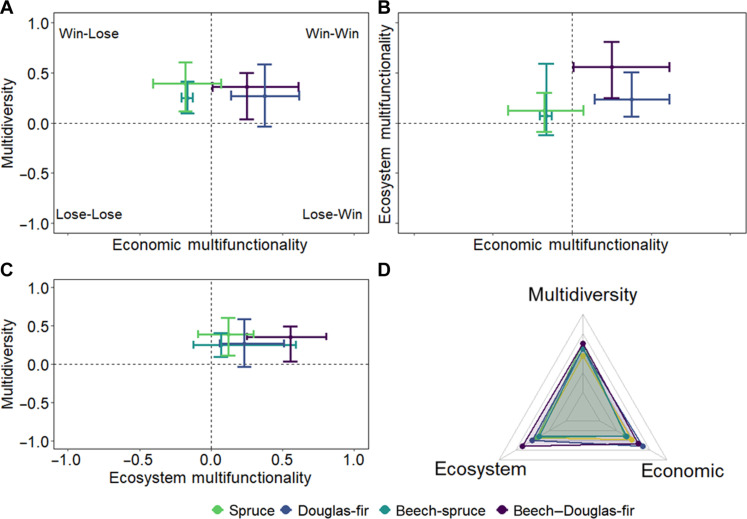
Effects of conifers, in mixture and pure stands, compared with pure beech stands on multidiversity, ecosystem multifunctionality, and economic multifunctionality. (**A** to **C**) Dots and lines (mean and 95% CI) represent proportional deviation in multidiversity, ecosystem multifunctionality, and economic multifunctionality resulting from enriching beech with conifers or pure coniferous stands. When 95% CI does not include zero, it indicates significant differences compared with pure beech stands. Values −1 and 1 indicate a 100% decrease or increase, respectively, in multidiversity, ecosystem multifunctionality, and economic multifunctionality compared to pure beech stands. (**D**) shows the average of multidiversity, ecosystem multifunctionality, and economic multifunctionality for each forest type (pure beech stands are represented in yellow).

Our results show significant trade-offs (i.e., win-lose) between multidiversity and economic multifunctionality only for beech-spruce mixture compared to pure beech stands (Fig. 4A). In this case, a significant increase in multidiversity came with a decrease in economic multifunctionality. Yet, this decrease in economic multifunctionality did not come at the expense of ecosystem multifunctionality, i.e., loss-neutral. In the case of beech–Douglas-fir mixture, we observed a significant win-win-win situation. A similar pattern was observed for pure Douglas-fir stands, but in this case, increases in ecosystem multifunctionality and economic multifunctionality (i.e., win-win) were not associated with significant increases in multidiversity compared with beech (win-neutral). Comparable trends were observed when using correlation among the three dimensions across forest types, in which multidiversity and ecosystem multifunctionality (*R*^2^ = 0.369, *P* = 0.02) and ecosystem and economic multifunctionality (*R*^2^ = 0.571, *P* < 0.001) were positively significantly correlated (fig. S7). In contrast, the correlation between multidiversity and economic multifunctionality was not significantly correlated (*R*^2^ = −0.025, *P* = 0.88).

## DISCUSSION

On the basis of a unique and comprehensive dataset and a three-dimensional multifunctionality concept, we show that the enrichment of European beech forests with commercial conifers can supply high levels of economic multifunctionality without compromising multidiversity and ecosystem multifunctionality. This indicates that adding a commercially important but functionally distinctive and potentially better climate-adapted species in tree species–poor forests can offer an important alternative for forest management. The positive effects of enrichment with Douglas-fir in contrast to spruce on ecosystem multifunctionality, i.e., nonadditive effects, point toward species-specific effects. Therefore, effects of enrichment need to be interpreted in the light of context-specific species selection. Overall, there were positive relationships between multidiversity and ecosystem multifunctionality, and between ecosystem multifunctionality and economic multifunctionality, but a neutral relationship between multidiversity and economic multifunctionality (fig. S7), suggesting potential contrasting mechanisms shaping ecosystem and economic multifunctionality. Compared to pure beech stands, beech–Douglas-fir mixture resulted in a win-win-win situation for the three dimensions. This outcome indicates that multipurpose forest management via enrichment of tree species–poor stands can provide habitat for different forest taxa and fulfill other ecological and economic goals.

### Understanding ecological and economic dimensions

The impact of forest type on single biodiversity indicators shifted along the forest’s vertical gradient, highlighting the relevance of including above- and belowground taxa to understand responses at the ecosystem level, via single biodiversity indicators, compositional changes, or multidiversity. Specifically, pure conifer stands, such as Douglas-fir stands, were associated with either neutral responses [e.g., Collembola and Oribatida (*62*)] or increases in belowground richness [e.g., soil and root fungi (*63*)]. This was accompanied by a decrease in species richness of canopy-associated groups, such as canopy beetles [fig. S3 and table S3, as previously reported (*26*, *64*–*66*)]. Yet, beech–Douglas-fir mixtures showed similar levels of species richness for canopy beetles as native beech forest stands (*26*, *64*–*66*). Thereby, enriching beech native forests with Douglas-fir may support belowground biodiversity and mitigate local biodiversity losses aboveground. While species richness and multidiversity can provide essential information, a more holistic perspective may require including other taxa (i.e., understory herbs and grasses, lichens and mosses, and deadwood-associated taxa), moving beyond richness by, for instance, understanding compositional changes (fig. S4) and considering forest specialist and conservation priority species [as discussed by Wildermuth *et al.* (*64*, *66*)]. Further, future research should also address the effects of stand structural diversity on integrative indexes of biodiversity (multidiversity) as evidence suggests that this is a key driver of biodiversity (*67*–*69*) and likely ecosystem multifunctionality (*14*, *70*) and economic multifunctionality. While this will improve the mechanistic understanding of the drivers of multifunctionality, it would likely not affect the synergies and trade-offs among multipurpose forest management dimensions.

Regarding single ecosystem functions and multifunctionality, significant positive diversity effects were primarily observed in the enrichment with Douglas-fir. Therefore, our results suggest that tree diversity benefits on ecosystem functioning (*23*, *31*, *58*, *71*, *72*) are also found in mixtures resulting from enrichment with nonnative species. Yet, we emphasize that careful consideration is needed before extrapolating our result to other nonnative species. Our results also highlight the need to consider the desired direction of the ecosystem function, which affects not only our study but also cross-studies interpretation of single ecosystem functions and the calculations of ecosystem multifunctionality. For example, litter decomposition responded strongly to forest type, with higher litter decomposition potentially interpreted as positive when aiming to increase nutrient turnover (as we considered here, see table S1) or negative when considering carbon storage (*73*). Moreover, drought resistance and pest vulnerability should be included when planning adaptive management actions, as well as indicators that capture potential negative loops (trade-offs), such as nitrate in groundwater resulting from forest management (*74*, *75*) and the effects of intraspecific variation in tree structure on wood quality (*76*).

Our study reveals the importance of including economic indicators reflecting multiple private forest management goals, as well as resistance to abiotic and biotic disturbances in trade-off analyses. In terms of profit-oriented goals, for example, several studies have already proven that pure coniferous stands economically outperform beech forests (*56*, *77*). This is especially true for Douglas-fir, which is on the rise as one of the most economically important nonnative tree species in Central European forests (*22*), together with others, such as Grand fir (*Abies grandis*) (*78*) and red oak (*Quercus rubra*) (*79*). Specifically, the high values of some indicators in Douglas-fir stands (i.e., immediate profit, medium-term income, and future timber production) result from intense harvesting and/or thinning predicted during the next 30 years as trees reach their target diameter (fig. S8). In contrast, pure beech stands have a longer production period due to their growth dynamics and take longer to reach the target harvest diameter. This difference in species development phases will lead to potential shifts in mixture composition, resulting in beech-dominated stands as the conifers in mixtures are harvested first (fig. S8). The indicator of resistance against abiotic and biotic disturbances also shows that mixed stands are a favorable alternative as tree survival of the more productive but also more susceptible conifers is increased in mixed forests (*40*, *59*, *60*). However, a major limitation of our study is the relatively short temporal scale considered, while important forest dynamics and successional phases are largely disregarded. Thus, our results must be interpreted as multifunctionality achievable in mature stands, while ranking might strongly differ in early stand development stages. As future development is crucial for economic considerations, we extended commonly used indicators like standing timber value by extensive forest growth simulation and valuation. However, considering the long-term management of European forests, it would be valuable to extend the economic snapshot provided by our study to a complete rotation period [as, e.g., in modeling approaches by Fuchs *et al.* (*56*)] and extend the monitoring of ecological functions and diversity in the plots over a longer period.

### Synergies between ecological and economic dimensions via enrichment with commercially important species

Our results show synergies (i.e., win-win-win situations) between multidiversity, ecosystem multifunctionality, and economic multifunctionality for beech forest stands enriched with Douglas-fir. This result supports growing evidence suggesting that beech forests in Central Europe can deal with a share of Douglas-fir without severe ecological or economic consequences (*57*, *80*), at least for mature stands. Thus, enrichment using functionally distinct and commercially important species is one promising option, particularly in scenarios where production-oriented objectives are among the main goals of forest owners. Alternatively, beech forests could be enriched with other species, such as oak species (*Quercus petraea* or *Quercus robur*) or Scots pine (*Pinus sylvestris*), which were not considered in our study. Native oak species, for example, are very important for biodiversity (*81*), hosting more phytophagous insects and forest specialists than beech (*82*). However, economically, oak has high associated establishment costs and long production periods (*83*), which often conflicts with conservation goals (*81*), making it a less viable option. Therefore, given our results, it is likely that these species (or other slow-growing or less productive species) might increase trade-offs among ecological and economic dimensions. Nevertheless, extending the species portfolio to further native and climate-adapted species is an essential line of research in the face of climate change (*20*). While we cannot directly extrapolate our results to a wider range of species combinations, our study contributes to the discussion on “novel mixtures” (*84*), which can serve as solutions to meet the increasingly diverse societal demands of forests and forest management.

Our results fill a knowledge gap by empirically showing a positive relationship between multidiversity and ecosystem multifunctionality in managed and mature forests. This relationship is not surprising when considering, for instance, the contribution of soil biodiversity to nutrient cycling, decomposition (*85*), and plant production (*86*); arthropods to decomposition, predation, and herbivory (*87*); or small mammals to seed dispersal, predation, and forest regeneration (*88*), among others. In addition, linkages between ecosystem multifunctionality and economic multifunctionality are exemplified by factors like tree biomass and the economic returns derived from timber sales (*39*, *89*). We observed a win-win situation between multidiversity and economic multifunctionality in beech–Douglas-fir mixture. This is notable, given that we did not include other direct and nonuse values of biodiversity (*90*), which are challenging to monetize (*39*, *91*), such as the recreational and cultural value of mixed forests (*92*, *93*). Yet, the relationship between multidiversity and economic multifunctionality may be affected by the set of indicators used to reflect economic functions. Therefore, our approach reflects economic goals through economic multifunctionality, representing the multitude of preferences from the private beneficiary perspective, which could be extended further.

While our emphasis here is on the stand level, which is the unit area where the management decisions are taken, previous research indicates that the positive impacts of mixtures on forest biodiversity can often be more pronounced at the landscape level (gamma diversity) across various species groups (*58*, *94*, *95*). However, positive effects of forest management on biodiversity at the landscape level were also previously achieved via a combination of single homogeneous management systems at intermediate spatial scales (8 to 18 ha) (*96*). Because of some taxa responding positively to within-stand heterogeneity and others to across-stand heterogeneity, heterogeneity at both stand and landscape levels is important (*96*). Combined with the observations of Schall *et al.* (*97*), such findings suggest that a mosaic of different forest types, including pure stands (*95*), at the landscape level, might be essential to promote regional biodiversity. Thus, promoting a diverse portfolio of forest types that offer complementary subsets of biodiversity and ecosystem functions is required to support multidiversity and intermediate levels of multifunctionality at the regional or landscape scale (*98*). Our study could form the basis for optimization approaches that combine multiple plots to account for larger spatial scales [see Neyret *et al.* (*46*)], using several indicators that represent different aspects of economic [instead of a few economic goals as in Fuchs *et al.* (*83*)] and ecological multifunctionality. This is pivotal when considering that, currently, Douglas-fir occurs at a relatively small spatial extent, for example, 4% of the area of the State Forests of Lower Saxony, where this study largely took place and a maximum of 10% in the long-term planning for the state forests of the study region (*99*), and 2% of the forest area in Germany (*51*), meaning that adverse effects and trade-offs might be enhanced in the case of larger coherent stands at the landscape level.

### Implications for forest management

In conclusion, bringing commercially valuable and climate resistance tree species into a matrix of native tree species does not necessarily reduce species richness or ecosystem functionality at the stand level. Our findings are highly relevant to forest management and policy in tree species–poor forests because they demonstrate that using nonnative species in a mixture—in moderate proportions, under a multipurpose forest management regime and following the principles of close-to-nature management—can promote reasonable levels of multidiversity, ecosystem multifunctionality, and economic multifunctionality. This might be particularly relevant for small forest owners with limited options to diversify their species portfolio at a larger spatial scale (*83*). Given that ecosystem functioning depends on the environmental context, e.g., Ratcliffe *et al.* (*31*), and that ideal species for enrichment can differ elsewhere, our study offers a roadmap that can and should be extended outside the studied region. Our findings contribute to the discussion of integrating biodiversity, ecosystem, and economic functions by providing empirical evidence for future forest management.

## MATERIALS AND METHODS

### Study area and study design

This study was conducted within the framework of the research training group (RTG) 2300, “Enrichment of European beech forests with conifers: impacts of functional traits on ecosystem functioning.” Study sites were located in mature, temperate state-owned forests across the federal state of Lower Saxony in Northwestern Germany. The 40 quarter-hectare study plots were grouped into quintets consisting of one plot of each of the five stand types (see the next paragraph), at eight different locations. Four quintets were established in the Solling and Harz mountains, with higher altitudes, lower mean annual temperatures, and higher annual precipitation than the other four sites established in the northern lowlands (*100*) (table S5). Besides these characteristics, the mountainous southern plots are characterized by Dystric Cambisols (*101*) that are nutrient rich and have a high clay content. In contrast, the northern lowland plots are characterized by Podzols, which have a low clay content and lower nutrient levels (*102*).

The quintets are composed of five plots representing different forest stand types: three pure plots (beech, Norway spruce, and Douglas-fir) and two beech-conifer mixtures (beech with Douglas-fir and beech with spruce). Each species in a mixture contributed ≥20% of the basal area, and ≥80% in pure stands at the moment of the plot establishment. Other coniferous and deciduous species, such as Scots pine (*P. sylvestris*), larch (*Larix decidua*), maple (*Acer* spp.), birch (*Betula pendula*), oak (*Q. petraea* and *Q. rubra*), and others, were also present in some plots, usually with a small basal area proportion.

### Data collection

The 40 plots were established in October 2017, but 7 were relocated in 2018 due to severe storm damage. Relocated plots were sited as close as possible in terms of both location and characteristics to the original plots, and this did not affect the overall results (via sensitivity analyses using only plots for which all variables were measured at the same location, fig. S9). Data from soil communities, soil and root fungi, soil carbon, nitrogen and N:C ratio, and microbial biomass were collected in 2017. Data from small mammals, birds, and root biomass were collected between 2018 and 2020. Because of storms and bark beetle outbreaks, four other plots were relocated in 2020. Data for natural tree regeneration, aboveground biomass, LAI, litter decomposition, canopy beetles, and the forest inventory used to calculate the economic indicators were collected in 2021. Two spruce plots lost in the summer of 2021 due to bark beetle outbreaks were excluded from the calculations (38 plots).

In total, species richness was assessed for nine biodiversity indicators, including Collembola, Oribatida, soil and root fungi [operational taxonomic units (OTU) richness], small mammals, tree diversity (understory tree species diversity), canopy beetles (species-level), canopy arthropods (OTU richness, assessed via metabarcoding), and birds (table S1). Nine ecosystem functions, including soil carbon (C) and soil nitrogen (N) stocks and N-to-C ratio, microbial biomass, aboveground biomass, LAI, litter decomposition, tree density (density of natural tree regeneration), and fine root biomass (table S1), and six economic functions (future wood volume, future timber production, medium-term income, immediate profit, immediate profit corrected by investment costs, and resistance against biotic and abiotic disturbances) were considered (table S2). However, some labor-intensive measurements were only performed on a subset of plots, and while included as single taxa (birds and canopy arthropods) and functions (fine root biomass), they were not included in the calculation of multidiversity or ecosystem multifunctionality across all plots but analyzed separately (figs. S3 and S5).

#### Soil communities—Collembola and Oribatida

Soil animals were sampled using a metal corer (5 cm diameter) between November 2017 and January 2018. One soil core was taken near the center of each plot and separated into litter (O_L_) and 0- to 5-cm and 5- to 10-cm soil depths, resulting in 120 samples (40 plots × 3 depths). Microarthropods were extracted using high gradient heat extraction. Collembola and Oribatida were then sorted and identified to species [for details, see Lu *et al.* (*62*)]. In our study, we combined species richness over litter and soil layers.

#### Root and soil fungi

The root and soil fungi were sampled between November and December 2017. Each plot was divided into four subplots, and five soil cores (8 cm diameter by 10 cm depth) were collected in three subplots (resulting in three replicates per plot). The samples were split between soil and root compartments. DNA from all samples and compartments were extracted and used for barcoding of fungal species as OTU by Illumina sequencing of the ITS region [see Likulunga *et al.* (*63*) and Rivera Pérez *et al.* (*103*) for more details].

#### Small mammals

Small mammal surveys took place between August and September 2018. At each plot, 64 Sherman traps were set in a grid of 10 × 10 m; all plots within each quintet were surveyed concurrently for four nights. Captured animals were identified to species and released at the point of capture [see detailed description of survey methods in Appleby and Balkenhol (*104*)]. Trapping and handling of small mammals were approved by the internal animal welfare committee of the University of Göttingen and conducted in compliance with the German Animal Welfare Act under the “Niedersächsisches Landesamt für Verbraucherschutz und Lebensmittelsicherheit” permit number 33.9-42502-04-18/2790. In our study, we focused on the species richness of small mammals derived from these surveys.

#### Canopy beetles

Between mid-April and mid-August 2021, three flight interception traps were placed in each plot in the tree canopies (average height = 17.5 m ± 3.1 m). The minimum distance between traps was 10 m. Traps consisted of a round lid (30 cm in diameter), two crossed window panes (50 × 24 cm), a funnel (26.5 cm in diameter), and a bottle (1 liter) with 200 ml of 50% polypropylene glycol. Each trap was emptied every 4 weeks, but samples were pooled per trap. Three samples were excluded from further analysis due to sample loss in at least one sampling period (two traps in Douglas-fir and one trap in European beech). More details can be found in Wildermuth *et al.* (*66*).

Taxonomic experts (W. Apfel, M. Hartmann, A. Kopetz, and A. Weigel) morphologically identified beetle individuals to species level. To avoid potential bias in stand-type comparisons due to short-term bark beetle calamities near some plots in the Harz mountains, all Scolytinae (bark beetles) were excluded from our dataset.

#### Natural tree regeneration

The natural tree regeneration of the plots was assessed during the summer of 2021 (May to June). The assessment consisted of six subplots of 10 × 10 m in all 40 plots, in which all the woody plant saplings equal to or higher than 50 cm in height were counted, and all seedlings higher than 80 cm were measured in terms of height. All seedlings were identified at the genus level, and, whenever possible, at the species level. The height was measured using a vertically graded measuring stick or a digital vertex for plants higher than 2 m, to provide precise information on the individual heights of the taller plants. The natural regeneration data were used as a proxy for understory plant diversity, and the number of saplings per hectare from 0.5 up to 1.60 m tall (i.e., density) was used to indicate tree recruitment.

#### Birds

Birds were surveyed with standardized 10-min point counts on five sampling dates for each plot in 2020. Point counts were conducted from the center of the plot by the same observer. The bird species seen and heard during the point counts and their abundances were listed within a 50-m radius. Double counting of individuals was avoided as much as possible by keeping track of heard and seen birds over the point count interval. The counts were done in the morning between sunrise and 11:00 a.m. in good weather conditions (i.e., avoiding strong winds and rain) [see Schuldt *et al.* (*26*) for further information].

#### Canopy arthropods (from metabarcoding)

Between the end of June and the end of July 2022, three flight interception traps were placed in the Solling forest plots (Dassel, Winnefeld, and Nienover) for 4 weeks in each plot. The traps and sampling positions used were the same as for canopy beetles (see above), but traps were filled with 99.5% polypropylene glycol. After collection, plant material was removed from the samples, which were stored in 96% undenatured, high-purity ethanol. For laboratory processing, sequencing (metabarcoding), and bioinformatics up until the assignment to OTUs, see Wildermuth *et al.* (*64*). To obtain arthropod taxon richness, all OTUs were assigned to taxa based on the BOLD database of European Arthropods (status: July 2022), using a similarity threshold of 97%. Only taxa with the species-level assignment and a minimum number of five reads per sample were considered for the analysis (*64*).

#### Soil carbon and nitrogen

Soil carbon and nitrogen were sampled from four points randomly chosen in all 40 plots. At each sampling point, the forest floor was collected using a steel frame (*d* = 28 cm) and sorted by layer. The layers were identified as litter (foliar), decay (nonfoliar), and humus (nonidentifiable and humidified). Mineral soil was also sampled using a core auger (*d* = 8 cm) and separated into layers at 0 to 5 and 5 to 10. The organic layer and mineral soil samples were dried at 60° and 40°C, respectively, until constant weight. For C and nitrogen (N) analysis, subsamples from the fine soil fractions (*d* < 2 mm) were ground with a mortar grinder RM 200 (Retsch, Germany) for 10 min. The organic layer samples were ball milled (MM2, Fa Retsch) for further chemical analyses. More detailed information is available in Foltran *et al.* (*102*). For the analyses in this paper, we used the aggregated information on carbon and nitrogen stock across the three organic soil layers (litter, decay, and humus) combined with the 0- to 10-cm depth of mineral soil.

#### Fine root biomass

The fine root sampling took place between March and April 2018, in four of the eight locations. A systematic regular sampling grid design of 10 × 10 m was used in each plot. Ten grid cells were systematically selected from the total 25 grid cells, and one root core was collected from each of them. A soil corer of 8 cm diameter was used to extract soil from both organic and mineral soil (from 0 to 60 cm of depth). Living roots were sorted by species based on morphology, due to the differences between beech and conifer roots [see Lwila *et al.* (*105*) for more details]. As the stand types did not include mixtures of the two conifer species, only the differentiation between beech and conifer roots was required. Roots from other tree species (<5%) in the research plots were excluded. The roots of less than 2 mm in diameter were classified as fine roots, and after sorting and processing, the living fine roots were expressed in terms of their biomass (*105*).

#### Aboveground biomass and Leaf Area Index (LAI)

We conducted a forest inventory in 2021 in all plots, where all trees with a Diameter at Breast Height (dbh) ≥ 7 cm were considered as the dominant stand layer. From these trees, the following information was recorded: species identity, dbh, geographical coordinates, and total height (for some of them). Both aboveground biomass and LAI were derived based on the forest inventory data from 2021. These two indicators of primary production were estimated following the allometric equations provided by Forrester *et al.* (*106*).

#### Microbial biomass

In each plot, three litter samples were collected on a transect 5 m apart between November 2017 and January 2018. Litter samples from each plot were pooled and were cut into pieces (<25 mm^2^). Microbial biomass was measured using substrate-induced respiration as described in Lu and Scheu (*25*). Briefly, maximum initial respiration response (MIRR) was determined as O_2_ consumption at 22°C 4 to 7 hours after addition of d-glucose, and microbial biomass (μg C_mic_ g^−1^) was derived as 38 × MIRR (*107*). Total carbon was determined using an elemental analyzer (*25*), and microbial biomass was expressed as per gram of carbon.

#### Litter decomposition

Freshly fallen litter was collected over 2 weeks in autumn 2018. This was done with mesh traps in the pure stands of European beech, Douglas-fir, and spruce in Solling (locations: Dassel and Winnefeld, table S5). The collected litter was dried at 60°C for 48 hours and then stored at 4°C. Each litter bag was filled with 5 g of either beech leaves, spruce needles, or Douglas-fir needles (one litter species per bag). Litterbags consisted of two different mesh sizes. For the bottom side, 0.5-mm mesh was used to keep needles from falling out. The top side 4-mm mesh was used to allow full access by soil animals. In 2019, litterbags were placed in the field in a grid of 3 m × 4 m and fixed with nails. After 24 months, samples were collected (randomly chosen from the grid). For transport, the litter bags were kept in LDPE bags and then stored at 4°C in the laboratory. Within 14 days, the litter material from the litterbags was cleaned from soil, moss, and ingrown roots, and exotic litter was picked out. Cleaned litter was weighed, freeze dried (VaCO2, GOT1000) in paper bags, weighed again, and stored at −20°C in a sealed paper bag. Litter decomposition was considered as the dry mass percentage after 24 months of experiment (2019 to 2021). We selected the litter samples to be the same as the tree species in the canopy (i.e., litter of beech in beech forests, and litter of beech and Douglas-fir in beech–Douglas-fir mixtures).

#### Economic indicators

*Rationale of selected economic functions*. For economic multifunctionality, we considered a range of economic functions, representing different economic goals from the perspective of a private beneficiary, i.e., forest owner or manager. The functions are then represented by indicators serving as proxies for these functions (see table S2). The economic functions were selected to represent different preferences toward time, risk, and management goals. Time preferences are reflected by two perspectives: the income that can be used from harvesting the stand immediately (economic function “immediate profit”) or the income from all expected revenues (minus costs) from leaving the stand but managing it through thinnings during the next 30 years (including valuation of final standing timber) (“medium-term income”). The 30-year projection period was chosen because during this period, the silvicultural focus will be on the current stands, while afterward, with continuing final harvests of trees that have reached the target diameter, establishing the next forest generation will gain importance. For this purpose, we used extensive bio-economic simulation (described below). These indicators were free of the risk of stand failure and represent a risk-neutral attitude. To account for risk aversion, we also considered the goal of minimizing the risk of stand failure (“resistance against abiotic and biotic disturbances”), given its high economic implications (*40*,*55*) and other severe effects on forest management goals (i.e., maintenance and provision of timber in the future and carbon storage). Further production-oriented management goals considered are the production of high-value wood, i.e., sawn wood products in the next 30 years (“future timber production”), as well as the wood volume at the end of the simulation period (“future wood volume”). The latter may reflect a “saving behavior” and aspects of sustainability maintaining future potential. While we focus on economic functions mimicking provisioning services, this indicator also reflects a management goal in which high wood volume for other ecosystem services is maintained (e.g., biodiversity or carbon), assuming that this might also generate income, e.g., through payments for ecosystem services, not explicitly quantified here. Thus, while the different economic indicators are largely based on the same dataset, the different indicators reflect distinct management goals.

*Bio-economic simulation and indicator calculation*. To calculate the indicators as proxies of economic functions (table S2), we used the forest inventory in 2021 (i.e., species, dbh, location, age, and height), representing the current state of the main stands. These data were used as input to project the forest development for the next 30 years in 5-year intervals (2021 to 2051, henceforth projection period) using the single-tree growth simulator WaldPlaner (*108*). Given that we look into future forest growth, we used available prediction on dynamic future site index accounting for a changing climate using a generalized additive model (GAM) [Schmidt (*109*)] using the representative concentration pathway (RCP) 8.5 and General Circulation Model (GCM) “Hadley Centre Global Environment Model” (HadGEM2) combined with the statistical regional “Wetterlagen-basierte Regionalisierungsmethode” (WettReg18) (*110*–*112*) (see Supplementary Methods S1).

The forest growth simulation is considered risk free; only mortality due to competition is regarded. The growth simulation generated data on standing and harvested wood volume and quadratic mean diameter of the trees at breast height, *QMD*, for 5-year time steps individually for beech, spruce, and Douglas-fir and combined for the other conifers and broadleaved species. Simulated data were used to directly derive the indicator “Future standing volume” and the standing wood volume at the end of the projection period (2051).

To convert wood volume into expected net revenues from timber harvesting, we used the *woodValuationDE* R package (*89*). The package incorporates the timber prices and harvest cost functions sensitive to tree species and *QMD* from Bodelschwingh (*113*). They are based on data from the forest administration of the Federal State of Hesse, in Germany, representing a typical situation for Central European forestry [more detailed information in woodValuationDE (*89*)]. For the function immediate profit, the standing wood volume was recalculated into net revenues from timber harvesting, assuming that the entire stand was immediately cut, which we refer to as “stumpage value.” The valuation approach (*89*) also allowed us to differentiate between different wood assortments, used for the indicator “sawn timber production.” It was calculated as the percentage share of the total harvested sawn timber volume from 2021 to 2051 per species using the *vol_assortment* function available in *woodValuationDE* [based on Offer and Staupendahl (*114*)], and then summed up for the projection period.

For the “annuity” as an indicator of medium-term income, we calculated the net revenues in each 5-year time step of the simulation period. On the basis of the annual net revenues, the net present value, *npv*, over the 30-year prediction period was calculated, assuming a positive time preference expressed by an interest rate of *i* = 0.015 (*115*). We calculated *npv* as the sum of present values of the net revenues of all harvests within the period plus the discounted change in the value of the standing trees (Eq. 1)

$$\mathrm{npv}=\sum_{t=0}^{t_{\max}}V_{h,t}{\left(1+i\right)}^{-t}+\left[V_{s,t_{\max}}{\left(1+i\right)}^{-t_{\max}}-V_{s,0}\right]$$ (1)

with the simulation time *t*, the duration of the prediction period *t_max_*, and the net cash flow *V_t_* at time *t*, [€ ha^−1^]; *V*_*h*,*t*_ refers to actual harvests and *V*_*s*,*t*_ refers to stumpage values of standing trees. We derived the *npv*’s annuity, *a* [€ ha^−1^ year^−1^] (Eq. 2), representing the annual average monetary success of forest management within the 30-year period, by

$$a=\mathrm{npv}\frac{i{\left(1+i\right)}^{t_{\max}}}{{\left(1+i\right)}^{t_{\max}}-1}$$ (2)

While the indicators described above focus on the short-term and the medium-term future economic development of the stands, we disregard past investments. To account for at least the most expensive past interventions, we included planting costs by compounding them to the present (2021; annual interest rate 0.015). Details of the adopted planting costs for each species are given in Supplementary Methods S2. We then reduced the immediate profit by the present value of the planting costs to obtain an investment-corrected economic indicator: “stumpage value corrected for planting costs,” as proxy for the economic function “Immediate Profit + investment costs.”

To account for risk attitudes and the management goal of reducing the economic risk of stand failure, we included the indicator “30-year tree survival probabilities” representing the function of resistance against abiotic and biotic disturbances, i.e., the ability of the stands to maintain crown cover by 2051. For that, we calculated (i) unconditional survival probabilities for each species, based on the survival time models by Brandl *et al.* (*60*), and then (ii) conditional stand-level survival probabilities, i.e., the probability of a stand to survive within the projected 30-year time period (2021 to 2051). The survival functions were estimated based on data from the European forest condition monitoring (levels I and II). The authors used an Accelerated Failure Time model assuming a two-parametric Weibull distribution of survival times. Survival probability depends on bioclimatic variables and the proportion of the species in the stand (*60*). We used the species proportion in 2021 and derived the bioclimatic variables from the WorldClim 1.4 dataset (*116*), for the period 2050 (average 2041 to 2060), GCM Hadgem2-ES, which includes the full Earth System configuration in its simulation (*111*). As we also assume that we look at the survival period of the 30-year simulation (projection period), we also used RCP 8.5 and the same GCM as for the growth simulations, but bioclimatic variables were taken from the WorldClim 1.4 dataset originally used for parameterizing the survival time models. The survival models are species specific and depend on the tree age and, for spruce and Douglas-fir, on the share of the species in the stand. We thus accounted for a stabilizing effect of the tree species mixture (fig. S11). Details on the equations used for these calculations are given in Supplementary Methods S3.

### Statistical analysis

We used the observed number of species or OTU as a measure of biodiversity for each individual taxon. For root and soil fungi, the OTU richness was rarefied using the function *rarefy* from the vegan package (*117*). For the biodiversity individual taxon, we checked for the shared percentage of species among forest types using the package ggVennDiagram (*118*) (fig. S4). First, we calculated correlation matrices (fig. S10) among the indicators using the corrplot package (*119*). Second, to calculate ecosystem multifunctionality, we first filled in six missing values in litter decomposition with the median value per forest type. Then, we standardized all the variables to unit scale (for biodiversity, ecosystem functions, and economic functions separately), using a normalization method (max-min), where all variables were transformed to a range of 0 to 1 (i.e., highest values would be transformed to 1, and the lowest values to 0, as we considered for all indicators “the more, the better”; tables S1 and S2). We then calculated multidiversity, ecosystem multifunctionality, and economic multifunctionality using the Hill-Chao approach correcting for correlation among the taxa/functions (*61*). We computed the area under the τ-curve (AUC) to obtain the integrative measures [see Chao *et al.* (*61*) for more details], and considered only the diversity of taxa/functions of *q* = 0, which represents the effective number of taxa/functions for each of the three dimensions. In addition, we calculated these integrative measures using the threshold approach (*16*, *120*). For the threshold approach, the results are displayed from 1 to 99% of the maximum observed values in our study (among the 38 plots) in fig. S2.

We analyzed the effects of forest type on (i) multidiversity, ecosystem multifunctionality, and economic multifunctionality and on (ii) the individual indicators (table S3 and figs. S3, S5, and S6) using generalized linear mixed-effect models via the package glmmTMB (*121*) (table S3 and Fig. 2). We used forest type as an explanatory variable, and quintets nested in the region were used as random effects to account for regional differences between quintets located in North and South (table S7). Because of convergence issues in models for single indicators, a simplified version of the random effect using only quintet was implemented for soil fungi, root fungi, LAI, microbial biomass, future wood volume, future timber production, medium-term income, immediate profit, and resistance against abiotic and biotic disturbances, and only region was used as a random effect for immediate profit corrected by investment costs. For the individual biodiversity indicators (except the rarefied fungi data), we included Poisson as the model family and also tested for data overdispersion using the package performance (*122*). We changed the model family to negative binominal (nbinom2) for canopy arthropods due to overdispersion. For all the other models, we used Gaussian distribution as the model family.

We tested the assumption of normality of the residuals in models based on diagnostic plots from the DHARMa package (*123*). If the normality test indicated a nonnormal distribution, the data were transformed using either log, square root, or cubic root transformations (the corresponding transformation is indicated in table S3—individual indicators). Then, we implemented the *glht* function of the multcomp package (*124*) using Tukey’s all-pairs comparisons, corrected by Bonferroni, to check the differences between forest types in multidiversity, ecosystem, and economic multifunctionality, and in the individual indicators. Model predictions and confidence intervals were obtained using the ggeffects package (*125*).

Further, we calculated net diversity effects using the proportional deviation (Eq. 3) approach suggested by Loreau (*126*)

$$\begin{array}{c} \mathrm{Dt}=\frac{\left[(Ot*pij)-\text{Etij})\right]}{\text{Etij}},\\ \text{Etij}=[(Eti*pi)+(Etj*pj)] \end{array}$$ (3)

in which *Dt* measures the proportional deviation of the observed species richness/provision level of a function (or multidiversity, ecosystem multifunctionality, or economic multifunctionality) of the mixture composed of *ij* species from its expected value (compared to the pure stands of the *ij* species). *Ot* is the observed level of function in a mixture and *pij* is the proportion (in terms of basal area) of species *ij* in the mixture. *Etij* is the expected level of a function in pure stands of *ij* species, represented by the provision level of a given function/taxa of species *i* weighted by its proportion in the mixture and the function level of species *j* weighted by its proportion in the mixture.

We assessed the effects of changing beech stands into enriched forests or coniferous pure stands (to simplify, in the calculations here, we consider that both represent a forest-type transformation) on multidiversity, ecosystem multifunctionality, and economic multifunctionality, calculating proportional deviation [see Martin *et al.* (*127*)]. The proportional deviation was calculated as follows

$$\text{Proportional deviation}=\frac{\text{forest transformation}-\bar{\text{beech stands}}}{\bar{\text{beech stands}}}$$

in which forest transformation refers to the value of multidiversity, ecosystem multifunctionality, or economic multifunctionality observed in the transformation option (here, either enrichment or pure coniferous stands), and $\bar{\text{beech stands}}$ is the average of pure beech stands used as the baseline. Values of −1 indicate a 100% decrease and values of +1 indicate a 100% increase in multidiversity, ecosystem multifunctionality, or economic multifunctionality compared to pure beech stands (Fig. 4).

For both net diversity effects and proportional deviation, we used the function boot from the boot package (*128*) in R to generate 10,000 ordinary nonparametric bootstrap replicates. The adjusted bootstrap percentile interval was obtained using the function “boot.ci” using 95% confidence level.

We also assessed trade-offs and synergies using Pearson’s correlations among multidiversity, ecosystem, and economic multifunctionality using the function ggpairs available in the GGally package (*129*) in R (fig. S7). All analyses were performed in R version 4.3.3 (*130*).

## Supplementary Materials

### This PDF file includes:

Figs. S1 to S11 Tables S1 to S5 Supplementary Methods S1 to S3 References
