# Supplementary material for: Enhancing economic multifunctionality without compromising multidiversity and ecosystem multifunctionality via forest enrichment
Source: Sci Adv. 2024 Oct 23;10(43):eadp6566. doi: 10.1126/sciadv.adp6566 (PMC11498224; doi:10.1126/sciadv.adp6566)
Supplement: Supplementary file 1 — Figs. S1 to S11 Tables S1 to S5 Supplementary Methods S1 to S3 References [file sciadv.adp6566_sm.pdf]

Science Advances
AAAS

Supplementary Materials for

Enhancing economic multifunctionality without compromising multidiversity and ecosystem multifunctionality via forest enrichment

Larissa Regina Topanotti *et al.*

Corresponding author: Larissa Regina Topanotti, larissa.topanotti@uni-goettingen.de

Sci. Adv. **10**, eadp6566 (2024)
DOI: 10.1126/sciadv.adp6566

This PDF file includes:

Figs. S1 to S11
Tables S1 to S5
Supplementary Methods S1 to S3
References

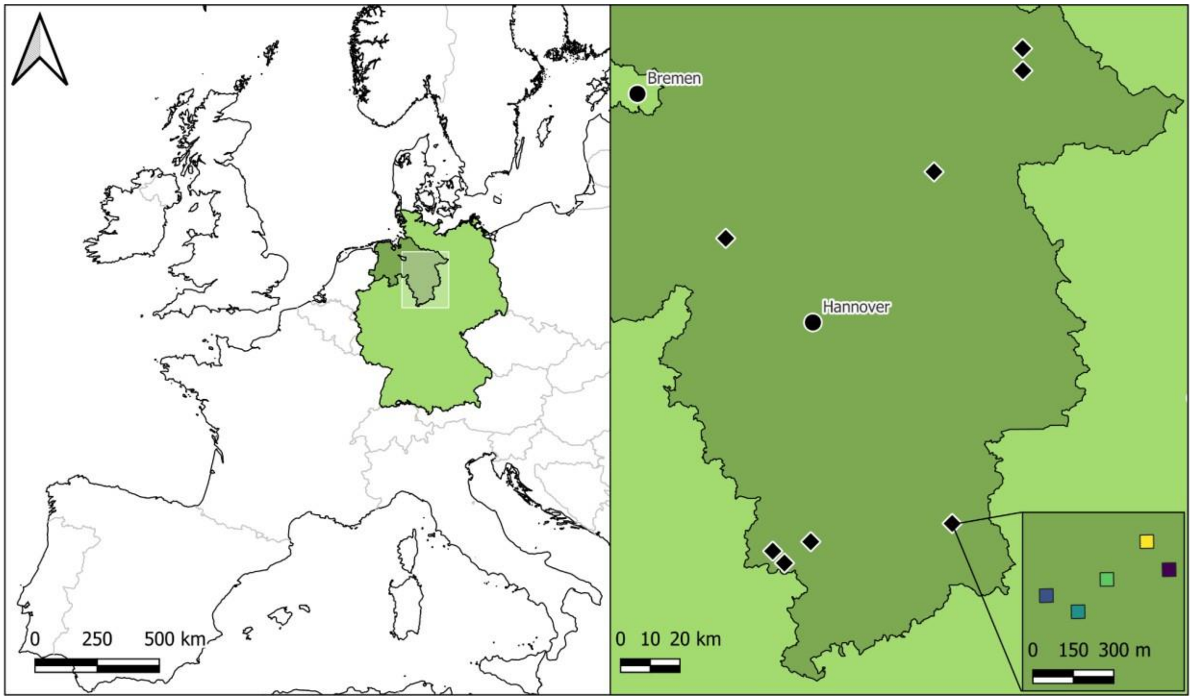

Fig. S1. Study area and location of the research plots (black diamonds) within the state of Lower Saxony (dark green) in Germany (light green). The lower right inset illustrates the arrangement of the plots in one of the 8 locations (in Harz mountains). The figure was adapted from Appleby and Balkenhol (*104*).

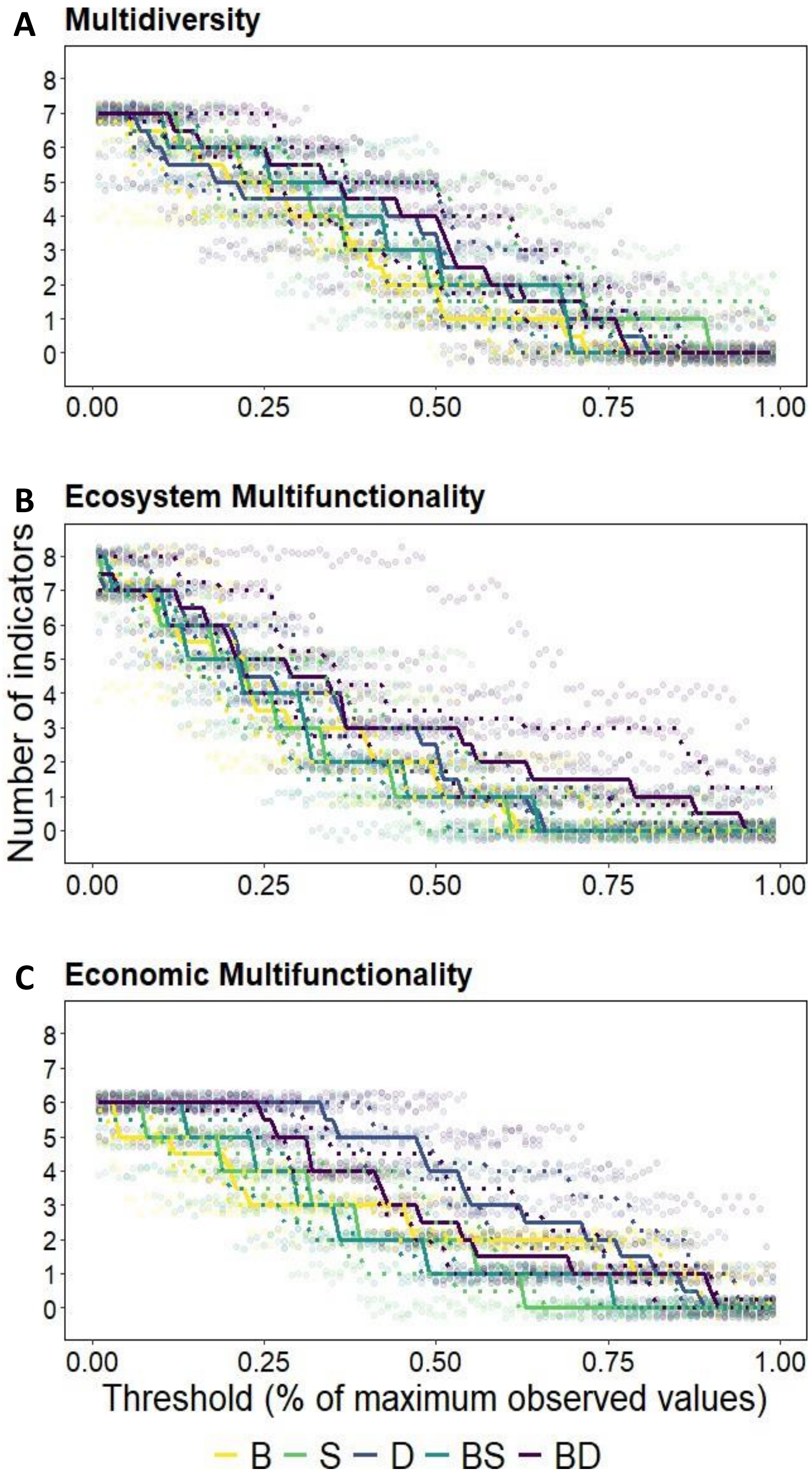

Fig. S2. Threshold approach of multidiversity, ecosystem multifunctionality, and economic multifunctionality across pure and enriched forests. Panels A-C show variation among forest types using the threshold approach, representing the number of indicators of each category (biodiversity and ecosystem and economic functions) that exceed a specific threshold. The dashed lines represent the quartiles, the bold solid lines represent the median for each forest type and the points are plot-level values. Forest types include beech forests (B), spruce forests (S), Douglas-fir forests (D), mixtures of beech with spruce (BS), and mixtures of beech.

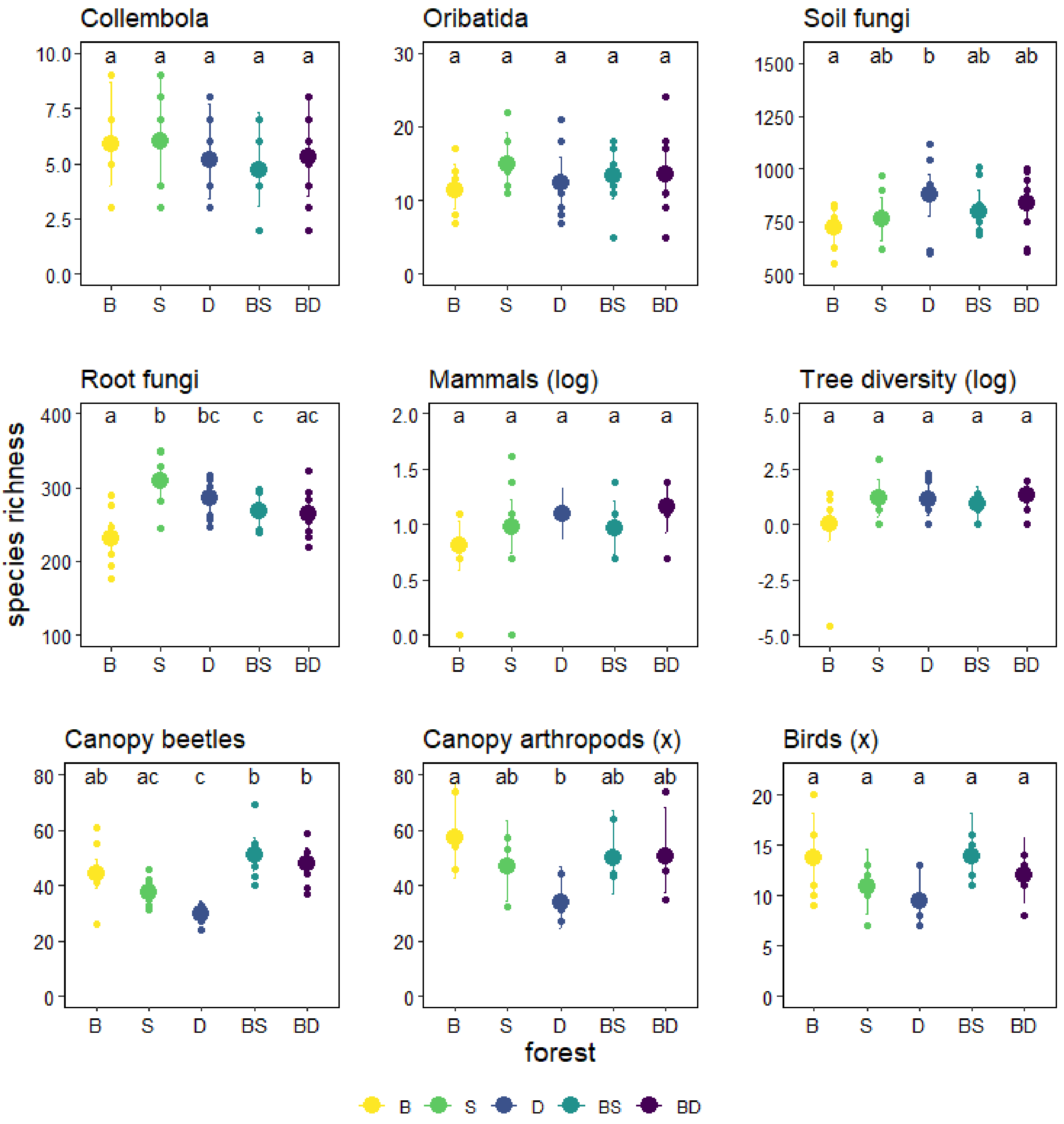

Fig. S3. Observed richness (species or OTU) of seven taxonomic groups across forest types. The graphs show the predicted values of species richness of seven taxa individually in beech forests (B), spruce forests (S), Douglas-fir forests (D), mixtures of beech with spruce (BS), and mixtures of beech with Douglas-fir (BD). Letters indicate significant differences between forest types based on pairwise Tukey's significance test ($p < 0.05$). An "(x)" indicates that the taxon was not included in the multidiversity analysis (Hill-Chao aproach). Data on small mammals, and natural regeneration were previously log-transformed to avoid significant problems in the residual dispersal (checked via DHARMa package). Regeneration refers to understory tree species diversity (Table S1).

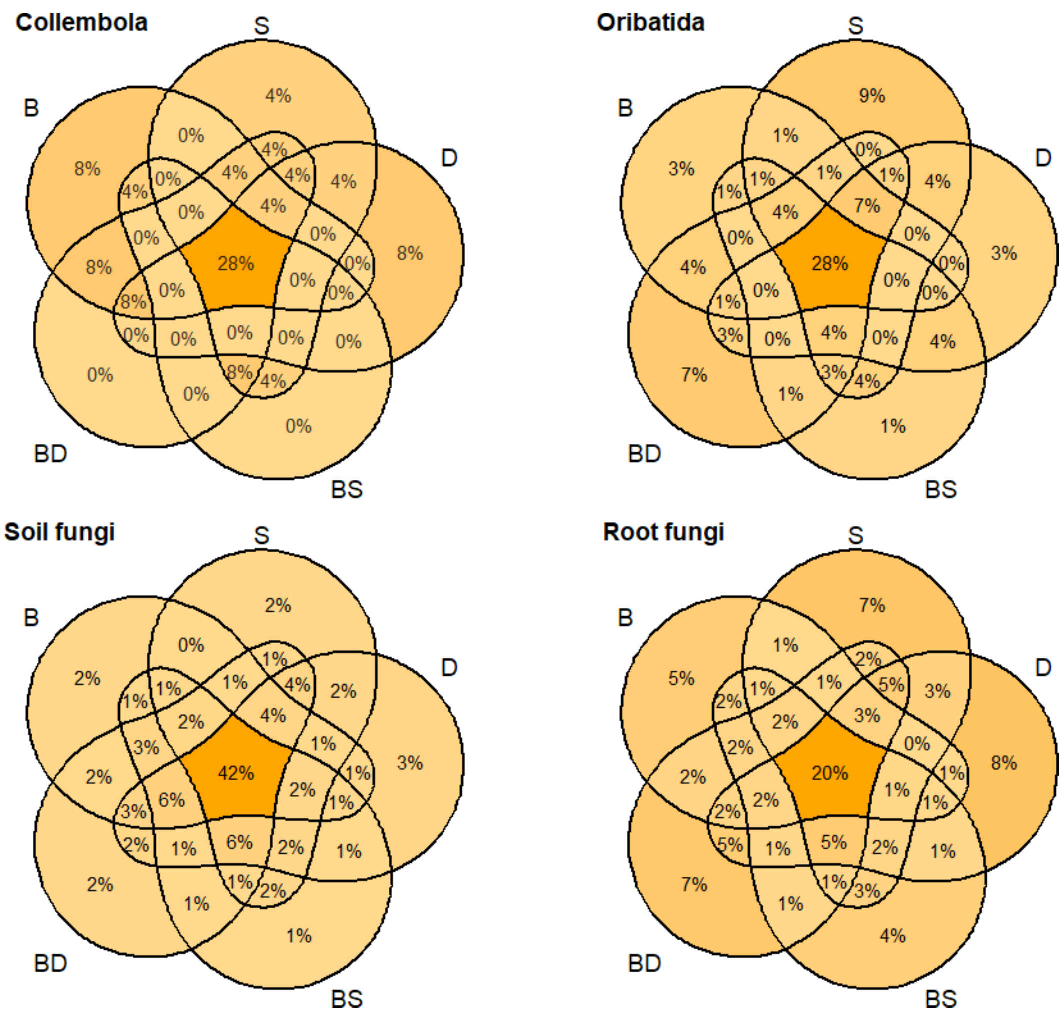
Collembola
Oribatida
Soil fungi
Root fungi
S
B
D
BD
BS

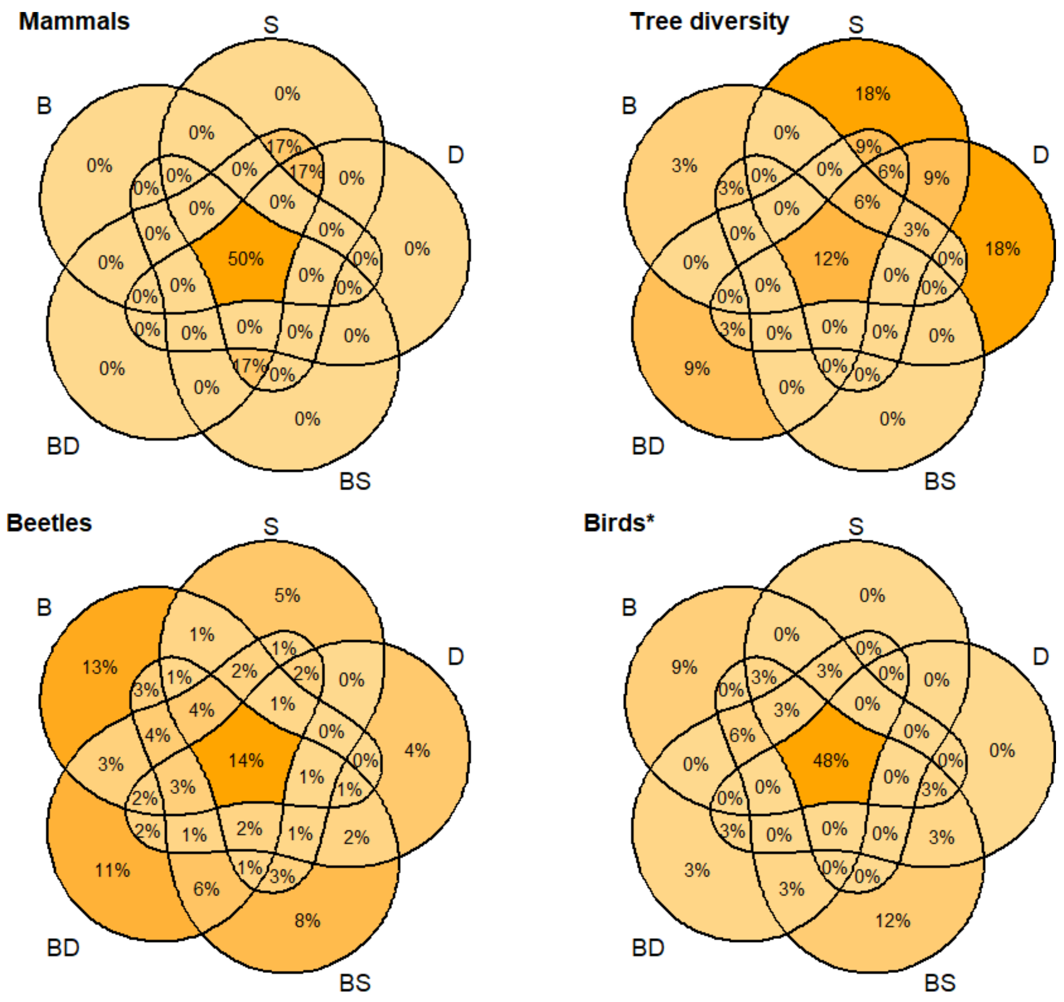

Fig. S4. Venn diagrams illustrating the shared and unique percentage of species/OTU across forest types. The graphs show the share of species that are individual to each forest type of shared among them. Forest type includes beech forests (B), spruce forests (S), Douglas-fir forests (D), mixtures of beech with spruce (BS), and mixtures of beech with Douglas-fir (BD). Birds* were not included in the calculation of multidiversity.

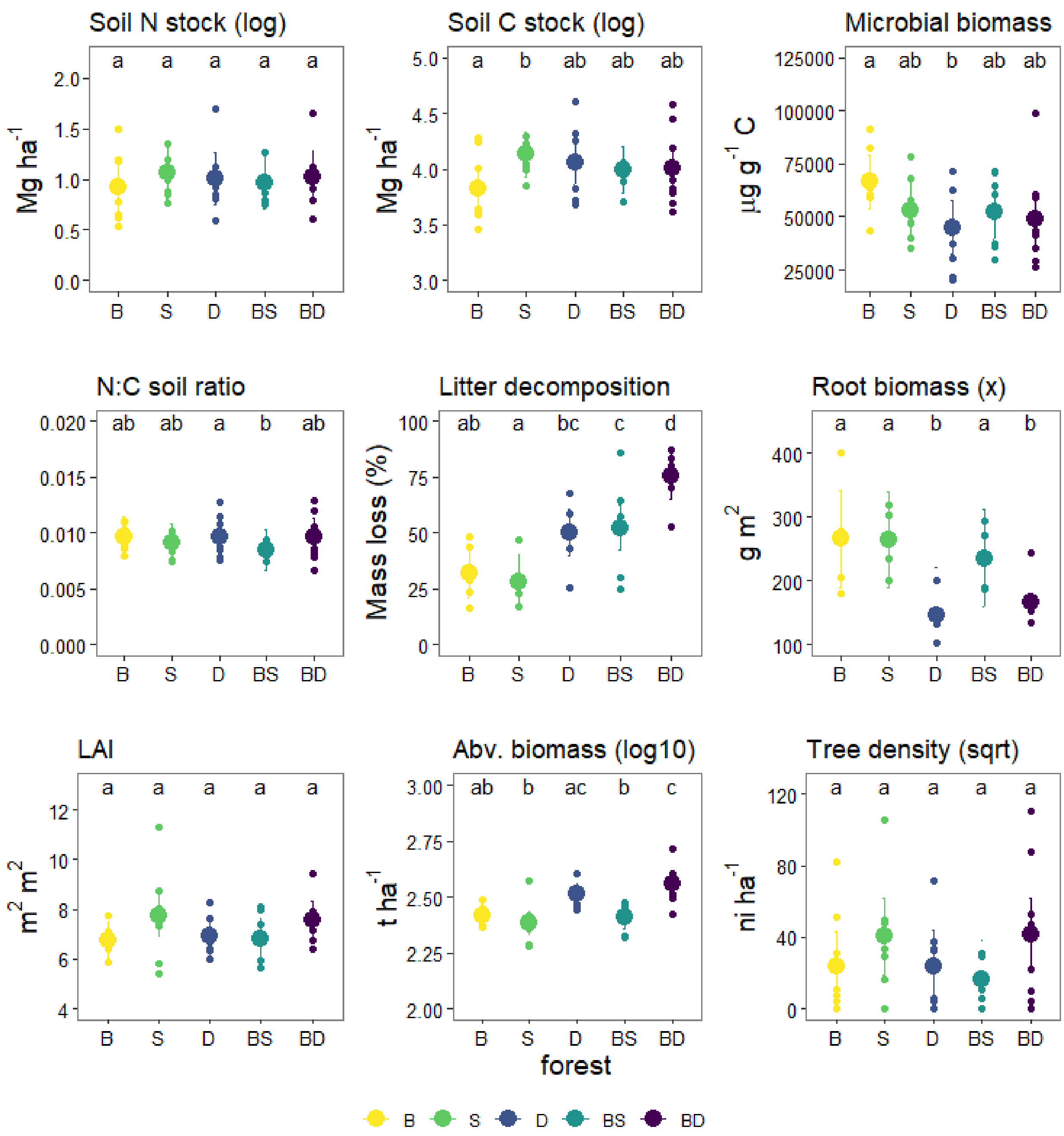

Fig. S5. Individual ecosystem functions across forest types. The graphs show the predicted values of nine ecosystem functions individually in beech forests (B), spruce forests (S), Douglas-fir forests (D), mixtures of beech with spruce (BS), and mixtures of beech with Douglas-fir (BD). Letters indicate significant differences between forest types based on pairwise Tukey's significance test ($p < 0.05$). An "(x)" indicates that the indicator was not included in the ecosystem multifunctionality analysis (Hill-Chao approach). Data on soil carbon and nitrogen stock were previously log-transformed, aboveground tree biomass was log10-transformed, and tree density was square-root transformed to avoid significant problems in the residual's dispersal (checked via DHARMa package).

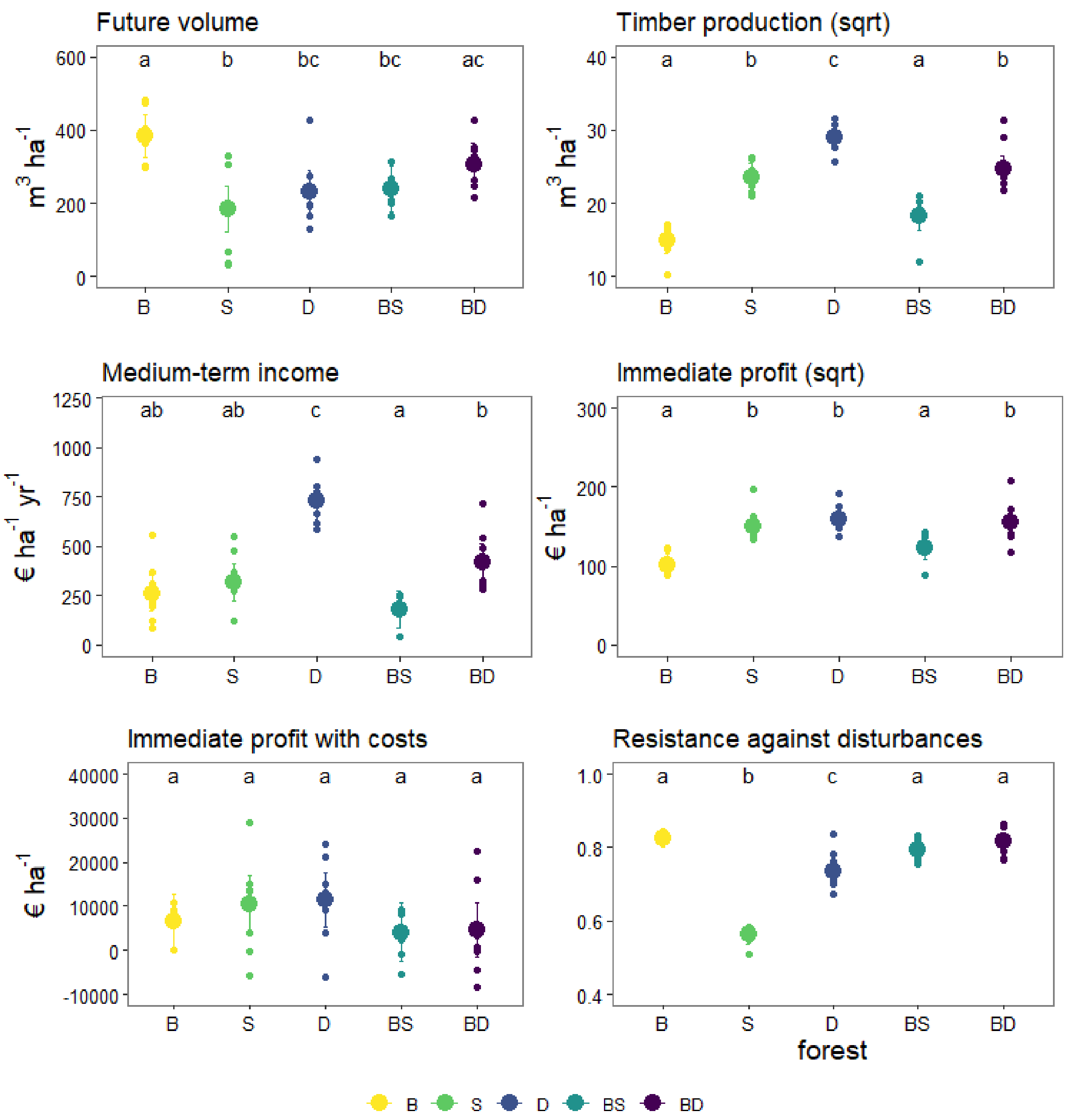

Fig. S6. Individual economic functions across forest types. The graphs show the predicted values of six economic functions individually in beech forests (B), spruce forests (S), Douglas-fir forests (D), mixtures of beech with spruce (BS), and mixtures of beech with Douglas-fir (BD). Letters indicate significant differences between forest types based on pairwise Tukey's significance test ($p < 0.05$). Data on timber production and immediate income were square-root transformed to avoid significant problems in the residual dispersal (checked via DHARMa package).

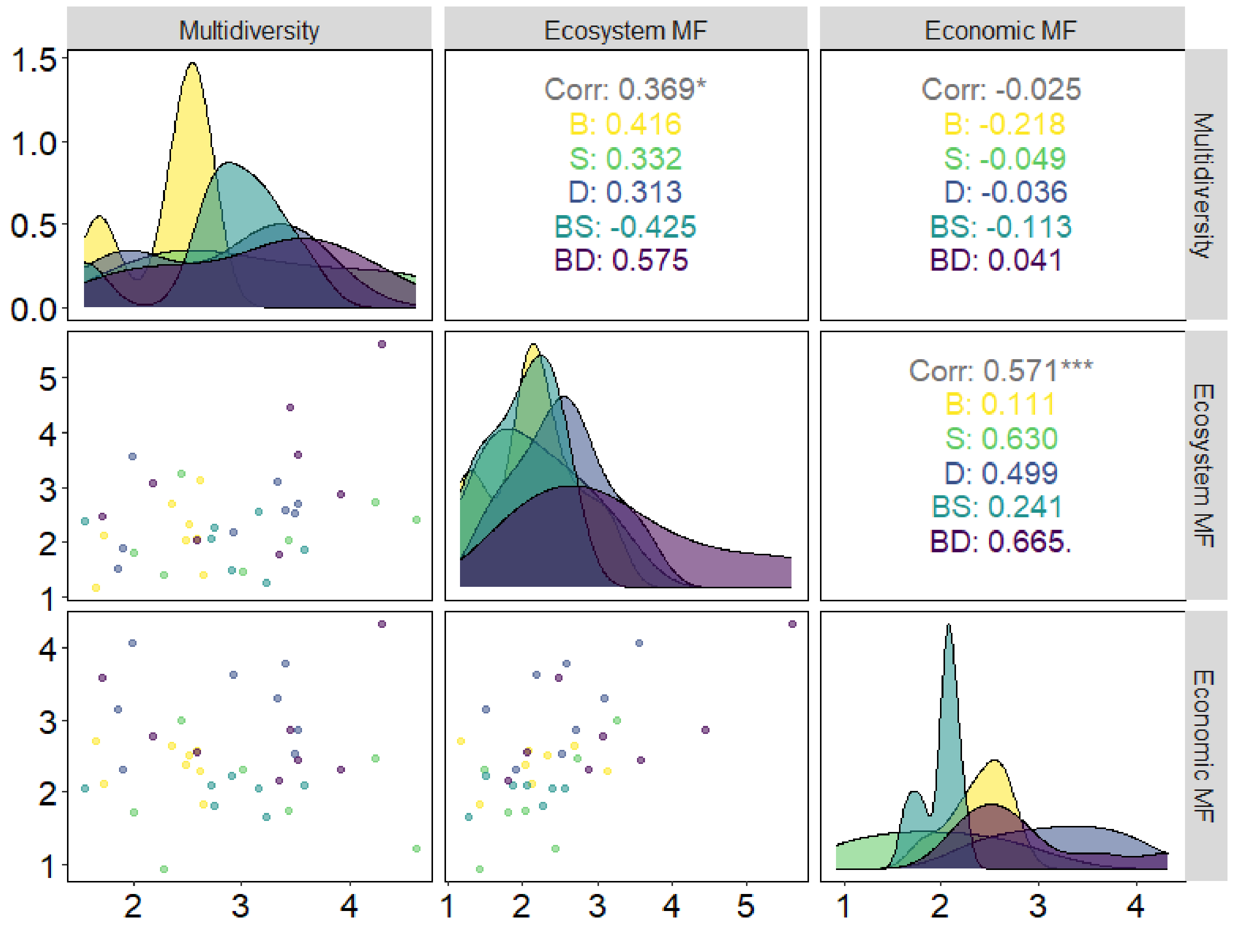

Fig. S7. Relationships between multidiversity, ecosystem multifunctionality and economic multifunctionality (MF) using Pearson correlation. The upper right part represents the correlations between multidiversity, ecosystem multifunctionality and economic multifunctionality (. p <0.1, * p < 0.05, ** p < 0.01, *** p < 0.001). The diagonal graphs represent the frequency of multidiversity, ecosystem multifunctionality and economic multifunctionality (number of plots) across the x-axis (values from 0 to 1). The lower left part shows the histograms between each two variables. Forest types include beech forests (B), spruce forests (S), Douglas-fir forests (D), mixtures of beech with spruce (BS), and mixtures of beech with Douglas-fir (BD).

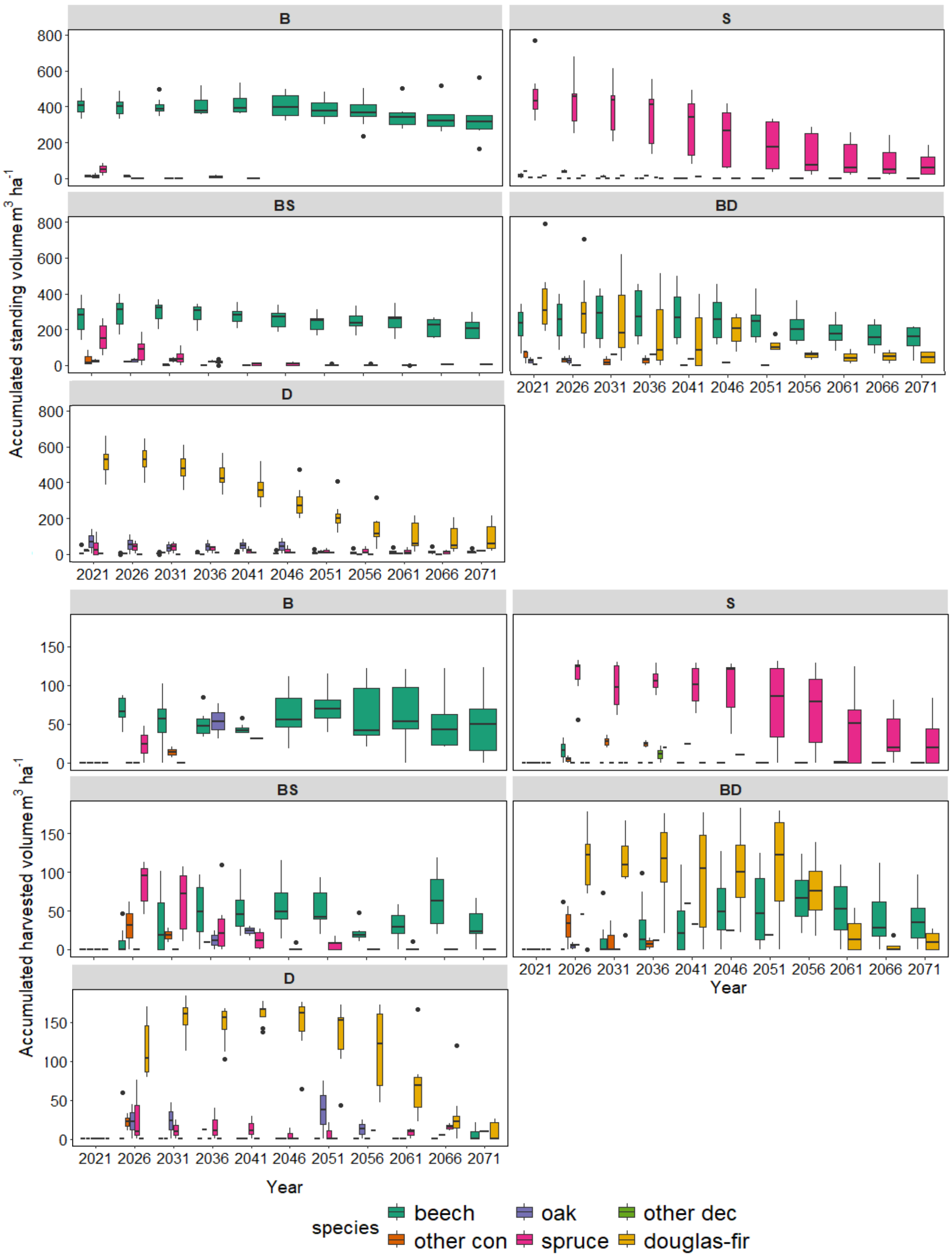

Fig. S8. Accumulated standing and harvested volume development over time for the different forest types. Results from the growth simulation show the accumulated (over each 5-year simulation step) standing and harvested volume for beech, Douglas-fir and spruce in pure and mixed stands. See Methods for details on the simulation procedure. Forest types include beech forests (B), spruce forests (S), Douglas-fir forests (D), mixtures of beech with spruce (BS), and mixtures of beech with Douglas-fir (BD). In the legend, "other dec" represents other deciduous species, "other con" represents other coniferous species and "oak" refers to *Quercus* spp. Note: here we are displaying the simulation results for 50 years (2021-2071) to illustrate the future development of the stands, especially the perspective of mixed stands turning into pure stands due to harvesting of the conifers. This period differs from our results displayed in the text when we used only 30 years (2021-2051).

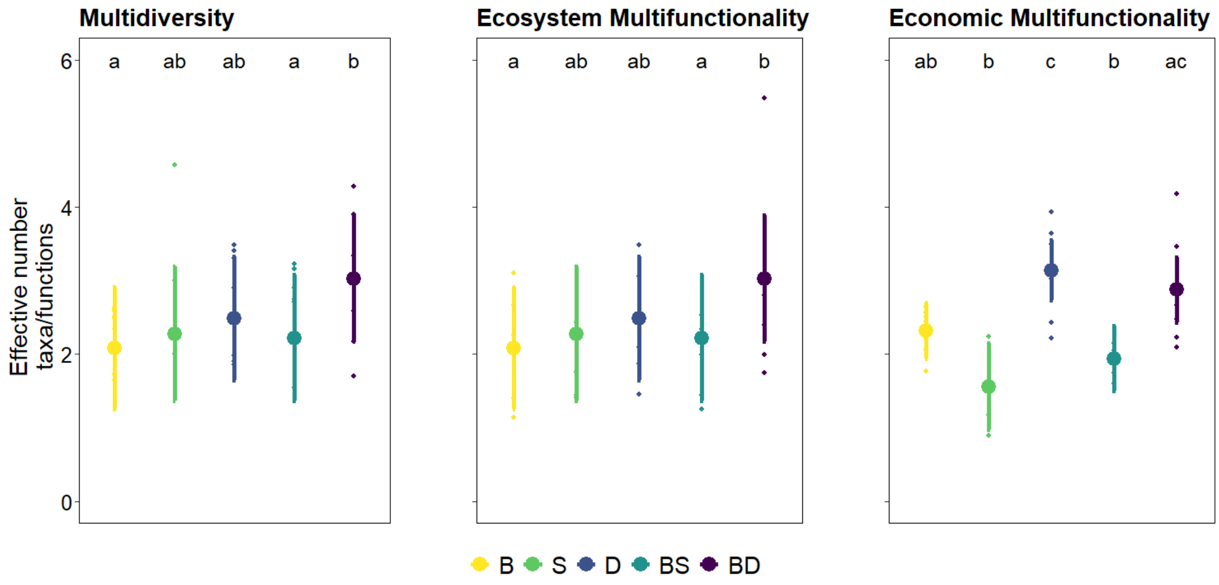

Fig. S9. Effects of forest type on the effective number of taxa/functions for multidiversity, ecosystem multifunctionality and economic multifunctionality (b) considering the common plots between 2021 and 2017/2018 (n = 31). This plot refers to the sensitivity analysis conducted for plot loss, to verify if there were differences in the multidiversity, ecosystem multifunctionality, and economic multifunctionality considering the data collected only in the same set of plots (more details in "Data Collection", in the main text). Forest types include beech forests (B), spruce forests (S), Douglas-fir forests (D), mixtures of beech with spruce (BS), and mixtures of beech with Douglas-fir (BD)

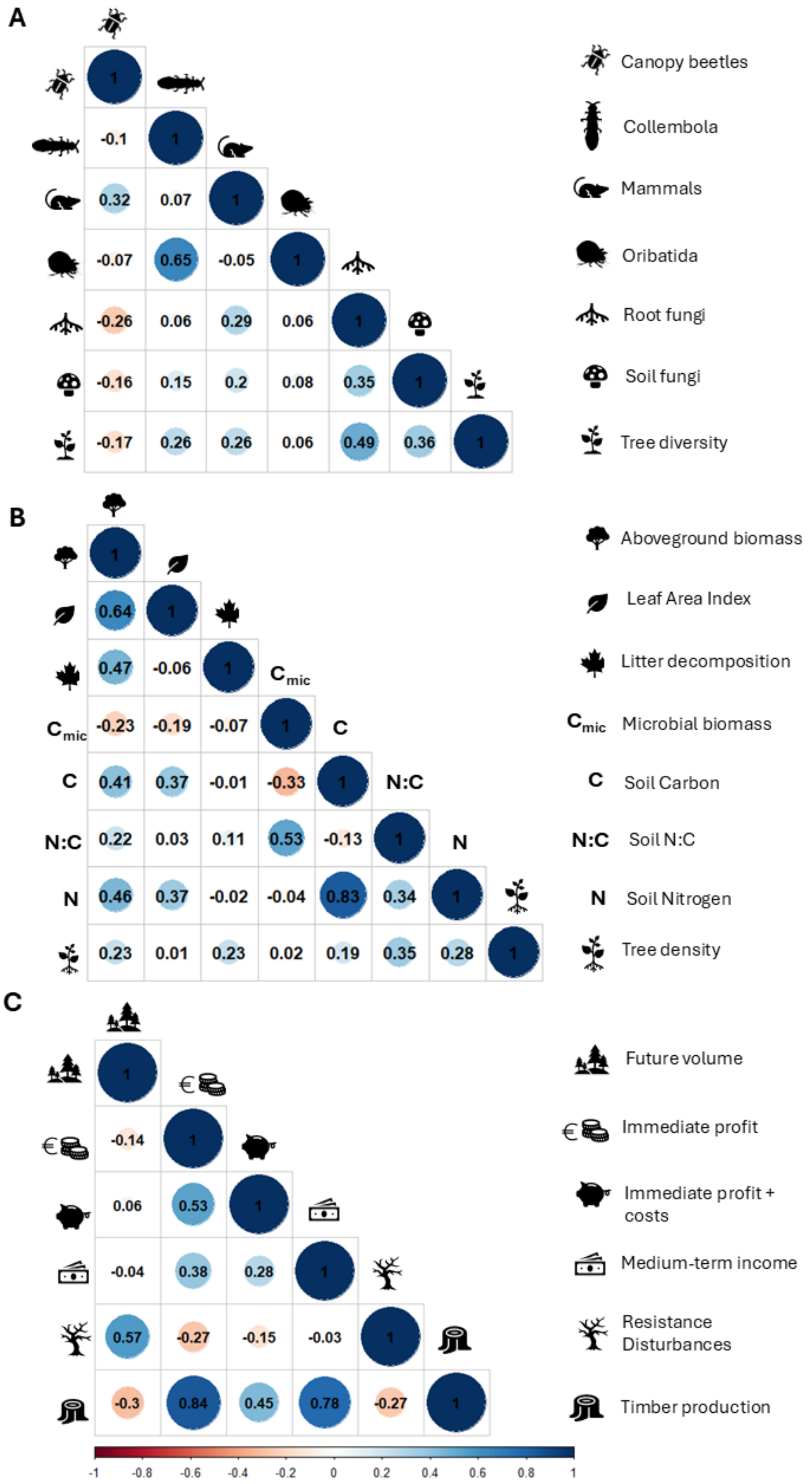

Fig. S10. Correlation matrices among the variables in each of the three dimensions of multipurpose forest management. A) indicators of multidiversity; B) indicators of ecosystem multifunctionality; C) indicators of economic multifunctionality.

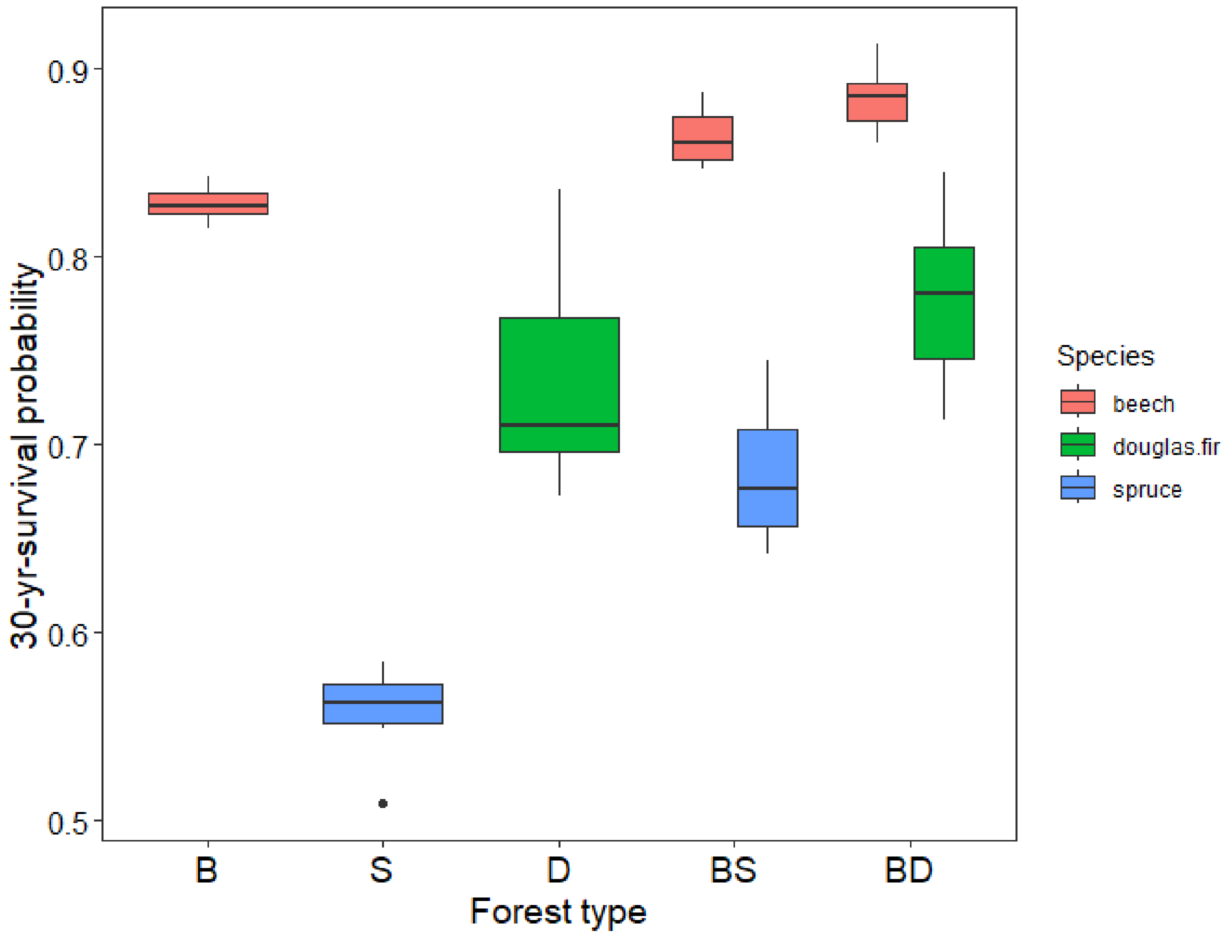

Fig. S11. 30-year-survival probabilities per species and forest type. The graph shows the survival probabilities (for the 30-year projection period) calculated for the main species (beech, Douglas-fir, and spruce) in each of the forest types under the representative concentration pathway (RCP) 8.5 using the General Circulation Model Hadgem, code HadGEM2 – ES, period of 2041-2060. For a detailed description of the calculation, see Methods. Forest types include beech forests (B), spruce forests (S), Douglas-fir forests (D), mixtures of beech with spruce (BS), and mixtures of beech with Douglas-fir (BD). The boxplots represent the variation in the 30-year-survival-probabilities for each species across the plot replicates of each forest type. The 30-year-survival probabilities were calculated only for the three main species: beech, spruce and Douglas-fir.

Function	Proxy/ Indicator	Description	Rationale	n
**Multidiversity**				
Biodiversity	Collembola	Species richness of soil Collembola (2017)	With our approach, we acknowledge the specific role that each group plays for specific ecosystem functions, but also the importance of higher species richness e.g. for food webs. Thus, we considered higher species richness across all groups to be better due to the intrinsic value of biotic diversity(*131*)	38*
	Oribatida	Species richness of soil Oribatida (2017)		38*
	Soil fungi	OTU richness of soil fungi (2017)		38*
	Root fungi	OTU richness of root fungi (2017)		38*
	Small mammals	Species richness of small mammals (2018)		38*
	Tree diversity	Species richness of plants in the understory community (0.5 m ≥ and dbh ≤ 7cm) (2021)		38*
	Canopy beetles	Species richness (2021)		38*
	Canopy arthropods	Taxon richness of canopy arthropods (2022)		15[x]
	Birds	Species richness of birds (2020)		29[x]
**Ecosystem multifunctionality**				
Nutrient and carbon cycling-related drivers	Soil nitrogen stock	Total nitrogen stock (Mg ha^{-1}) in the forest floor combined with mineral soil (0-10 cm) (2017)	Here we consider that high nitrogen stocks represent a continuous source of plant-available N(*132*)	38*
	Soil carbon stock	Total carbon stock (Mg ha^{-1}) in the forest floor combined with mineral soil (0-10 cm) (2017)	Carbon is directly connected to soil fertility as it is one of the elemental constituents of humus(*133*). In addition, carbon stock in the soil contributes to carbon sequestration(*134*).	38*
	Microbial biomass	Microbial carbon (µg g^{-1} C) in the litter layer (O_L) (2017)	Microbial biomass influences the formation of humus and soil organic matter(*135*, *136*), fueling the soil food webs(*137*) . A higher microbial biomass would mean greater nutrients available to trees and energy available to the decomposer communities.	38*
Nutrient cycling-related processes	Soil N:C ratio	Soil N:C ratio in forest floor combined with mineral soil (0-10 cm) (2017)	The soil C: N ratio is important to determine the soil microbial community structure, for example(*138*). It is also an important indicator of nutrient leaching(*132*)	38*
	Litter decompositi on	Litter mass loss (%) of the same species as the canopy after 24 months (2021)	Litter decomposition contributes to nutrient cycling processes. Here, we consider “the more the better” for litter decomposition, because litter decomposition not only facilitates nutrient availability to plants(*139*, *140*) but also long-term soil organic matter formation in temperate regions(*73*)	32*
Primary plants production	Fine root biomass	Total biomass of living fine root in forest floor and 0-10 mineral soil layer combined (g m²) (2018)	Fine roots are important for acquiring soil resources and mediating biogeochemical cycling(*141-143*)	20 [x]

	Leaf mass	Leaf Area Index (LAI, m² m²) (2021)	The leaf area index is connected to physical and biological processes in the canopies, especially energy fluxes exchange(*144*, *145*). We assume that a higher LAI indicates a greater capacity for photosynthesis.	38*
	Aboveground biomass	Total aboveground biomass of all trees (t ha^{-1}) (2021)	Aboveground tree biomass is highly correlated with the amount of carbon sequestered by trees(*146*). Tree stems can store most of the total carbon trees store (~ 50%)(*147*, *148*). Greater aboveground biomass represents greater amounts of carbon stocked in the trees.	38*
Tree density	Natural tree regeneration density	Number of saplings per hectare (density) of natural regeneration from 0.5 m up to 1.60 m tall (2021)	A higher density of natural tree regeneration represents a higher capacity for recovery after disturbances and a high capacity for succession, decreasing costs with the establishment of future tree generation(*149*)	38*

Table S1. Individual indicators of multidiversity and ecosystem multifunctionality from pure and mixed forests of beech with Douglas-fir and spruce in Lower Saxony, Germany. Note: n represents the number of plots where the data were collected; OTU: operational taxonomic units; the year of data collection/estimation for each indicator is shown in parenthesis; indicators followed by * were considered to calculate the estimated number of taxa/functions and threshold approaches of either multidiversity or ecosystem multifunctionality, accordingly. In these calculations, all indicators were considered in the direction of "the more the better". Indicators followed by an x were not included in this calculation due to the smaller sample size (canopy arthropods and birds for multidiversity, and fine root biomass for ecosystem multifunctionality).

Function	Proxy/ Indicator	Description	Rationale	n
Future wood volume	Future standing volume	Standing volume at the end of the projection period (2051) ($m^3\ ha^{-1}$)	Expected stand development over 30 years - period when the main silvicultural focus will be on the current stands	38
Future timber production	Sawn timber production	Total of sawn wood extracted from the harvested volume ($m^3\ ha^{-1}$) during the projection period (30 years, 2021-2051)	The potential of the standing and ingrowing wood volume to be transformed into long-living/high-value usage products (i.e. furniture or construction wood)	38
Medium-term income	Annuity	Net present value (*npv*) annuitized with the interest rate over the projection period (30 years, 2021-2051), (€ $ha^{-1}\ yr^{-1}$)	Annual average monetary success of the forest management - it represents the economic potential of each forest, considering both the costs and revenues from final harvesting and thinning operations in the next 30 years.	38
Immediate Profit	Stumpage value	Current net revenue of the standing timber (2021) (€ ha^{-1})	Current economic wood value; net revenue from the forests if they were harvested now.	38
Immediate Profit + investment costs	Stumpage value corrected for planting costs	Current net revenue of standing wood reduced by the compounded planting costs (€ ha^{-1})	Current economic potential is reduced by the compounded initial costs (planting). If the forests were naturally regenerated, there would be no planting costs. Planting costs were compounded with the stand age.	38
Resistance against abiotic and biotic disturbance	30-year-tree-survival probabilities	Tree survival probabilities for the projection period (2021-2051) weighted by the species proportions in each plot (%)	Risk of stand failure estimated by survival functions showing the probability that a species reaches the year 2051. Similar to the conditional failure probability within a time step, or the hazard rate, as suggested by[111], we derived the conditional probability that the stands survive until the end of the projection period (next 30 years)	38

Table S2. Individual indicators of economic multifunctionality from pure and mixed forests of beech with Douglas-fir and spruce in Lower Saxony, Germany. All indicators were considered to calculate the average and threshold approaches of economic multifunctionality. All indicators were considered in the direction of “the more the better”.

	Response variables	forest type $\mathcal{X}^2$	Df	Pr (>$\mathcal{X}^2$)
	Multidiversity	10.351	4	**0.03491***
	Ecosystem Multifunctionality	26.4	4	**<0.001***
	Economic Multifunctionality	36.826	4	**<0.001***
**Multidiversity**	Collembola	1.5406	4	0.8194
	Oribatida	3.8445	4	0.4275
	Soil fungi	13.798	4	**0.0079***
	Root fungi	36.422	4	**<0.001***
	Small mammals (log transformed)	7.8295	4	*0.09803.*
	Canopy beetles	52.098	4	**<0.001***
	Tree diversity (log transformed + 0.01)	9.084	4	*0.0590.*
	Canopy arthropods (metabarcoding)	13.527	4	**0.0088***
	Birds	6.1922	4	0.1852
**Ecosystem multifunctionality**	Soil nitrogen stock (log transformed)	4.8578	4	0.3022
	Soil carbon stock (log transformed)	11.619	4	**0.0204***
	Microbial biomass	13.099	4	**0.0108***
	N:C ratio	11.43	4	**0.0221***
	Litter decomposition	51.369	4	**<0.001***
	Fine root biomass	45.403	4	**<0.001***
	Leaf Area Index	5.7482	4	0.2188
	Aboveground tree biomass (log 10 transformed)	35.805	4	**<0.001***
	Tree density (square root transformed)	5.0348	4	0.2837
**Economic multifunctionality**	Future wood volume	32.398	4	**<0.001***
	Future timber production (square root transformed)	162.05	4	**<0.001***
	Medium-term income	89.157	4	**<0.001***
	Immediate profit (square root transformed)	54.043	4	**<0.001***
	Immediate profit with planting costs	5.5731	4	0.2334
	Resistance against disturbances	428.46	4	**<0.001***

Table S3. Generalized linear mixed model (GLMM) analyzing the effect of forest type on multidiversity, ecosystem multifunctionality and economic multifunctionality as well as the individual indicators of each of these dimensions. Multidiversity included Collembola, Oribatida, soil fungi, root fungi, small mammals, canopy beetles and tree diversity (understory tree species diversity). Ecosystem multifunctionality included soil nitrogen stock, soil carbon stock, microbial biomass, soil N:C ratio, litter decomposition, leaf area index, aboveground biomass, and tree density. Although canopy arthropods and birds were not included in the calculation of multidiversity as well as fine root biomass for ecosystem multifunctionality, the effects of forest type on these indicators are also shown here. Economic multifunctionality was comprised of future wood volume, future timber production, medium-term income, immediate profit, immediate profit with plantings costs and resistance against abiotic and biotic disturbances. The p-values (Pr ($\mathcal{X}^2$)) are based on type III Qui-square tests ($\mathcal{X}^2$) through glmmTMB::Anova, package glmmTMB. An asterisk follows significant effects at 95% probability (highlighted in bold), and a dot follows (marginal) significant effects at 90% probability (highlighted in italics). Data on small mammals, tree regeneration, birds, soil nitrogen, soil carbon, aboveground tree biomass, tree recruitment, sawn timber production and current stumpage value were previously transformed (specified in the table) to avoid significant problems in the residuals´ dispersal (checked via DHARMa package). Df = degrees of freedom.

Dimension		Forest type<br>B	S	D	BS	BD
**Multidiversity**	predicted	2.32	3.16	2.80	2.86	3.12
	95% CI	[1.81, 2.83]	[2.62, 3.70]	[2.29, 3.31]	[2.32, 3.39]	[2.61, 3.63]
	Gain (%)	-	36.0	20.7	23.3	34.5
**Ecosystem MF**	predicted	2.12	2.32	2.51	2.14	3.24
	95% CI	[1.29, 2.95]	[1.47, 3.17]	[1.68, 3.34]	[1.29, 2.99]	[2.41, 4.08]
	Gain (%)	-	8.6	18.4	0.9	52.4
**Economic MF (sqrt transformed)**	predicted	2.37	1.86	3.17	2.00	2.84
	95% CI	[1.98, 2.79]	[1.50, 2.26]	[2.72, 3.66]	[1.63, 2.41]	[2.42, 3.30]
	Gain (%)	13.2	-	30.1	3.7	24.3

Table S4. The effective number of taxa or functions predicted values of multidiversity, ecosystem multifunctionality, and economic multifunctionality on pure and mixed forest stands of beech, spruce, and Douglas-fir and their confidence intervals. Predictions are based on generalized linear mixed models. Forest types include beech forests (B), spruce forests (S), Douglas-fir forests (D), mixtures of beech with spruce (BS), and mixtures of beech with Douglas-fir (BD). Data on the effective number of functions for economic multifunctionality was previously square-root transformed to avoid significant problems in the residuals´ dispersal (checked via DHARMa package. Note: the gain line represents the percentage of increase in the other forest types compared to the forest type with the minimum value observed in each dimension.

Forest Type	Stem density	Basal area	Prop (%)	Age	Elev (m a.s.l.)	Slop e (°)	MAP (mm yr^{-1})	MAT (°C)	Sand (%)	Silt (%)	Clay (%)
Harz (South)											
B	468	30.43	100	104	524	19.3					
S	40	4.11	—	99	511	13.6					
D	216	45.46	98.3	54	520	15.8	1029	7.6	16	16	68
BS	200	16.65	—	99	507	13.7					
BD	236	54.21	17.1;82.9	104	492	15.2					
Dassel (South)											
B	484	28.3	100	91	442	3.7	822.9	8.6			
S	412	57.92	100	89							
D	376	40.89	96.6	45	362	2.1	814.9	8.7	26	53	21
BS	396	25.21	80.5;19.5	91; 81	442	3.7	822.9	8.6			
BD	356	41.91	49.4;38.7	92;90	442	2.2	822.9	8.6			
Winnefeld (South)											
B	252	30.6	96.4	93	379	3.8	818.2	8.9			
S	628	42.55	100	57							
D	208	35.39	99.4	48	375	4.8	839.3	8.8	20	57	23
BS	221	27.48	74.4;25.6	98;90	345	6.2	818.2	8.9			
BD	175	32.06	61.5;38.5	93;83	368	7.9	818.2	8.9			
Nienover (South)											
B	576	30.89	98.3	90	320	8.3	869.9	9.1			
S	312	37.06	100	58	393	7.1	895.4	8.8	20	57	23
D	248	38.23	81.6	48	405	3.7	895.4	8.8			
BS	508	38.18	41.0;59.0	71;64							
BD	208	38.88	47.8;52.2	130;127							
Nienburg (North)											
B	420	29.93	97.7	81	101	0.3					
S	240	30.13	86.5	64	84	1.6					
D	264	38.61	96.8	64	88	0.8	733.3	9.7	80	13	7
BS	272	33.16	74.8;20.7	81;80	98	2.7					
BD	332	40.97	29.9;70.1	110;111	89	4					
Unterlüß (North)											
B	524	27.99	93.5	88	162	2					
S	252	31.2	91.2	114	149	0.5					
D	276	40.09	85.4	73	167	0.9	746.6	9	79	15	6
BS	332	36.1	52.7;37.8	125	162	0.9					
BD	416	36.66	47.9;41.9	88;88	166	1.7					
Göhrde II (North)											
B	380	35.24	79.8	99	117	3.6					
S	492	36.69	95.1	59	140	0.8					
D	372	37.31	84.3	56	128	1.1	681.7	9.2	79	15	6
BS	400	38.55	49.4;50.6	120;97	138	1					
BD	672	37.35	18.4;54.6	69;56	126	0.6					
Göhrde I (North)											
B	356	25.97	95.0	133	115	4.4					
S	348	40.83	96.7	64	121	2.1					
D	256	40.53	93.0	56	126	0.6	672.6	9.2	73	24	3
BS	416	31.04	38.3;39.4	83	113	2.8					
BD	302	42.32	28.2;71.8	77;56	125	0.3					

Table S5. Characteristics of the forest stands of beech, Douglas-fir and Norway spruce and the mixtures of beech with Douglas-fir and beech with spruce in the state of Lower Saxony, Germany. Note: stem density = number of stems ha^{-1}; basal area = m² ha^{-1}; prop (%) = percentage of each species in terms of basal area in 2021 in either their monocultures or mixtures; in case of a mixture, the first value refers to beech and the second to the respective conifer; age refers to the

main species only (beech, Douglas-fir and spruce), in 2021; in the mixtures, the first age refers to beech and the second to the conifer component; if there is only one number, they have the same age according to the forest record books; elevation interpolated from SRTM digital elevation model; MAP: mean annual precipitation (average from January 1981 to December 2018); MAAT: mean annual temperature (average from January 1981 to December 2018). Source: Ammer et al.(*100*); Foltran et al. (*102*). Forest types include beech forests (B), spruce forests (S), Douglas-fir forests (D), mixtures of beech with spruce (BS), and mixtures of beech with Douglas-fir (BD).

Supplementary Methods

S1 Simulation of future forest growth development

Future forest development in terms of wood volume of different species and diameter classes was derived from the single-tree growth simulator WaldPlaner(*108*) for the next 30 years in 5-year intervals (2021 - 2051) (see Methods section in main manuscript). We accounted for a changing climate by predicting the site index, one major variable in the simulator's growth functions, based on soil, climate, and nitrogen deposition data using a generalized additive model (GAM) as described in Schmidt(*109*). The predictor variables temperature sum in the growing season, precipitation sum in the growing season, and nitrogen deposition were averaged over the stands' lifespan and the respective projection period of the 5-year intervals. Thus, the site index was updated after each 5-year projection. Climate data is projected under the representative concentration pathway (RCP) 8.5 using the General Circulation Model (GCM) 'Hadley Centre Global Environment Model' (HadGEM2) combined with the statistical regional '*Wetterlagen-basierte Regionalisierungsmethode*' (WettReg18)(*110-112*). This represents a rather pessimistic climate projection but the difference in the effect on site index in these mature forests is insignificant compared to a rather optimistic climate projection from the global 'European Centre Earth System' model (EC-EARTH12) combined with the dynamical 'Regional Atmospheric Climate Model' (RACMO)(*150,151*). Thus, we refrained from running the growth simulations with climate ensembles. Thinning and harvesting decisions were simulated according to the regional management guidelines of a graduated thinning from above considering a prescribed basal area(*152*).

S2 Planting costs assumed for each species

While the indicators of future standing volume, sawn timber production, annuity, and stumpage value (Table S2) focus on the stands' current and medium-term future economic development, we disregard past investments. However, precise information on the past thinning and harvesting operations conducted in the stands was missing. To account for at least the most expensive past interventions, we included planting costs by compounding them to the present (2021; annual interest rate 0.015). For that, we retrieved information on whether the species were planted and the species´ age from forest management records (*Bestandeslagerbuch*) of the state forest. To summarize, almost all the beech trees were naturally regenerated, while most of the spruce and all Douglas-fir stands were planted. To account for these differences in establishment costs, we considered the planting costs (where applicable) adopted by Fuchs et al.(*56*), that is, € ha^{-1} 3,000 for beech, € ha^{-1} 2,625 for spruce and € ha^{-1} 4,400 for Douglas-fir. We did not assume any costs for natural regeneration (mostly for beech in either pure or mixed stands), even though we know that tending costs might accrue. We then reduced the immediate profit by the present value of the planting costs to obtain an investment-corrected economic indicator: 5) immediate profit reduced by compounded planting costs.

S3 Detailed calculation of the "resistance against abiotic and biotic disturbances" via tree survival probabilities

The 30-year-survival probabilities of the individual tree species (Eq. S1) were calculated as:

$$\breve{S}_{s,p,w_{s,p}}(30yr) = 1 - \left[\frac{S_{s,p,w_{s,p}}(t_{s,p,2021}) - S_{s,p,w_{s,p}}(t_{s,p,2051})}{S_{s,p,w_{s,p}}(t_{s,p,2021})}\right] \qquad \text{[Eq. S1]}$$

with the conditional survival probability $\breve{S}_{s,p,w_{s,p}}(30yr)$, which represents the conditional probability that the species s at plot p with the basal-area share of the species in the stand $w_{s,p}$ survives until the end of the projection period (next 30 years), the (unconditional) survival probability of the tree species from successful establishment of the stand until the inventory in 2021 $S_{s,p,w_{s,p}}(t_{s,p,2021})$ based on its age according to the inventory $t_{s,p,2021}$ and its unconditional survival probability until the year 2051 $S_{s,p,w_{s,p}}(t_{s,p,2051})$. Results from this equation for the individual species are shown in Fig S11.

We were interested in the probability that the crown/tree cover of a stand would be maintained within the next 30 years rather than the probability of losing a specific tree species due to disturbances. For mixed-species stands, we thus derived the stand-level 30-year-survival probabilities as the average of the species' 30-year-survival probabilities weighted by their basal area (Eq. S2). For instance, for a stand with two species s_1 and s_2, the stand-level 30-year-survival probability is:

$$\breve{S}_{stand,p}(30yr) = \breve{S}_{s_1,p,w_{s_1,p}}(30yr) \cdot w_{s_1,p} + \breve{S}_{s_2,p,w_{s_2,p}}(30yr) \cdot w_{s_2,p} \qquad \text{[Eq. S2]}$$

Results from this equation (Eq. S2), for each research plot, are displayed in the “resistance against abiotic and biotic disturbances” indicator, in Fig S6.

REFERENCES AND NOTES

1. FAO, Global Forest Resources Assessment 2020—Main report (2020). https://doi.org/10.4060/ca9825en.

2. European Commission, New EU Forest Strategy for 2030—Communication from the Commission to the European Parliament, the Council, the European Economic and Social Committee and the Committee of the Regions. (2021).

3. P. Hildebrandt, P. Kirchlechner, A. Hahn, T. Knoke, R. H. Mujica, Mixed species plantations in Southern Chile and the risk of timber price fluctuation. *Eur. J. For. Res.* **129**, 935–946 (2010).

4. A. Lindh, M. K. Sundqvist, E. P. Axelsson, N. J. Hasselquist, F. X. Aguilar, D. Alloysius, U. Ilstedt, Functional traits to predict financial value of enrichment planting in degraded tropical forests. *New For.* **55**, 1283–1310 (2024).

5. C. Sabogal, E. de Almeida, D. Marmillod, J. O. P. Carvalho, *Silvicultura na Amazonia Brasileira: Avaliação de experiências e recomendações para implementação e melhoria dos sistemas* (CIFOR, 2006).

6. A. S. Forbes, K. J. Wallace, H. L. Buckley, B. S. Case, B. D. Clarkson, D. A. Norton, Restoring mature-phase forest tree species through enrichment planting in New Zealand’s lowland landscapes. *N. Z. J. Ecol.* **44**, 1–9 (2020).

7. D. C. Zemp, A. Gérard, D. Hölscher, C. Ammer, B. Irawan, L. Sundawati, M. Teuscher, H. Kreft, Tree performance in a biodiversity enrichment experiment in an oil palm landscape. *J. Appl. Ecol.* **56**, 2340–2352 (2019).

8. M. L. Hobi, C. Ginzler, B. Commarmot, H. Bugmann, Gap pattern of the largest primeval beech forest of Europe revealed by remote sensing. *Ecosphere* **6**, 1–15 (2015).

9. T. H. Durrant, D. de Rigo, G. Caudullo, *Fagus sylvatica* and other beeches in Europe: Distribution, habitat, usage and threats, in *European Atlas of Forest Tree Species* (Publication Office of the European Union, 2016).

10. E. Ampoorter, L. Baeten, M. Vanhellemont, H. Bruelheide, M. Scherer-Lorenzen, A. Baasch, A. Erfmeier, M. Hock, K. Verheyen, Disentangling tree species identity and richness effects on the herb layer: First results from a German tree diversity experiment. *J. Veg. Sci.* **26**, 742–755 (2015).

11. E. Ampoorter, L. Barbaro, H. Jactel, L. Baeten, J. Boberg, M. Carnol, B. Castagneyrol, Y. Charbonnier, S. M. Dawud, M. Deconchat, P. D. Smedt, H. D. Wandeler, V. Guyot, S. Hättenschwiler, F.-X. Joly, J. Koricheva, H. Milligan, B. Muys, D. Nguyen, S. Ratcliffe, K. Raulund-Rasmussen, M. Scherer-Lorenzen, F. van der Plas, J. V. Keer, K. Verheyen, L. Vesterdal, E. Allan, Tree diversity is key for promoting the diversity and abundance of forest-associated taxa in Europe. *Oikos* **129**, 133–146 (2020).

12. D. C. Zemp, N. Guerrero-Ramirez, F. Brambach, K. Darras, I. Grass, A. Potapov, A. Röll, I. Arimond, J. Ballauff, H. Behling, D. Berkelmann, S. Biagioni, D. Buchori, D. Craven, R. Daniel, O. Gailing, F. Ellsäßer, R. Fardiansah, N. Hennings, B. Irawan, W. Khokthong, V. Krashevska, A. Krause, J. Kückes, K. Li, H. Lorenz, M. Maraun, M. S. Merk, C. C. M. Moura, Y. A. Mulyani, G. B. Paterno, H. D. Pebrianti, A. Polle, D. A. Prameswari, L. Sachsenmaier, S. Scheu, D. Schneider, F. Setiajiati, C. A. Setyaningsih, L. Sundawati, T. Tscharntke, M. Wollni, D. Hölscher, H. Kreft, Tree islands enhance biodiversity and functioning in oil palm landscapes. *Nature* **618**, 316–321 (2023).

13. F. Van Der Plas, P. Manning, E. Allan, M. Scherer-Lorenzen, K. Verheyen, C. Wirth, M. A. Zavala, A. Hector, E. Ampoorter, L. Baeten, L. Barbaro, J. Bauhus, R. Benavides, A. Benneter, F. Berthold, D. Bonal, O. Bouriaud, H. Bruelheide, F. Bussotti, M. Carnol, B. Castagneyrol, Y. Charbonnier, D. Coomes, A. Coppi, C. C. Bastias, S. Muhie Dawud, H. De Wandeler, T. Domisch, L. Finér, A. Gessler, A. Granier, C. Grossiord, V. Guyot, S. Hättenschwiler, H. Jactel, B. Jaroszewicz, F.-X. Joly, T. Jucker, J. Koricheva, H. Milligan, S. Müller, B. Muys, D. Nguyen, M. Pollastrini, K. Raulund-Rasmussen, F. Selvi, J. Stenlid, F. Valladares, L. Vesterdal, D. Zielínski, M. Fischer, Jack-of-all-trades effects drive biodiversity–ecosystem multifunctionality relationships in European forests. *Nat. Commun.* **7**, 11109 (2016).

14. A. Sanaei, A. Ali, Z. Yuan, S. Liu, F. Lin, S. Fang, J. Ye, Z. Hao, M. Loreau, E. Bai, X. Wang, Context-dependency of tree species diversity, trait composition and stand structural attributes regulate temperate forest multifunctionality. *Sci. Total Environ.* **757**, 143724 (2021).

15. X. Li, H. Wang, J. Luan, S. X. Chang, B. Gao, Y. Wang, S. Liu, Functional diversity dominates positive species mixture effects on ecosystem multifunctionality in subtropical plantations. *For. Ecosyst.* **9**, 100039 (2022).

16. P. Manning, F. Van Der Plas, S. Soliveres, E. Allan, F. T. Maestre, G. Mace, M. J. Whittingham, M. Fischer, Redefining ecosystem multifunctionality. *Nat. Ecol. Evol.* **2**, 427–436 (2018).

17. A. Paquette, C. Messier, The role of plantations in managing the world's forests in the Anthropocene. *Front. Ecol. Environ.* **8**, 27–34 (2010).

18. E. G. Brockerhoff, L. Barbaro, B. Castagneyrol, D. I. Forrester, B. Gardiner, J. R. González-Olabarria, P. O. Lyver, N. Meurisse, A. Oxbrough, H. Taki, I. D. Thompson, F. Van Der Plas, H. Jactel, Forest biodiversity, ecosystem functioning and the provision of ecosystem services. *Biodivers. Conserv.* **26**, 3005–3035 (2017).

19. L. Depauw, E. De Lombaerde, E. Dhiedt, H. Blondeel, L. Abdala-Roberts, H. Auge, N. Barsoum, J. Bauhus, C. Chu, A. Damtew, N. Eisenhauer, M. V. Fagundes, G. Ganade, B. Gendreau-Berthiaume, D. Godbold, D. Gravel, J. Guillemot, P. Hajek, A. Hector, B. Hérault, H. Jactel, J. Koricheva, H. Kreft, X. Liu, S. Mereu, C. Messier, B. Muys, C. A. Nock, A. Paquette, J. D. Parker, W. C. Parker, G. B. Paterno, M. P. Perring, Q. Ponette, C. Potvin, P. B. Reich, B. Rewald, M. Scherer-Lorenzen, F. Schnabel, R. Sousa-Silva, M. Weih, D. C. Zemp, K. Verheyen, L. Baeten, Enhancing tree performance through species mixing: Review of a quarter-century of TreeDivNet experiments reveals research gaps and practical insights. *Curr. For. Rep.* **10**, 1–20 (2024).

20. J. Wessely, F. Essl, K. Fiedler, A. Gattringer, B. Hülber, O. Ignateva, D. Moser, W. Rammer, S. Dullinger, R. Seidl, A climate-induced tree species bottleneck for forest management in Europe. *Nat. Ecol. Evol.* **8**, 1109–1117 (2024).

21. E. Pötzelsberger, H. Spiecker, C. Neophytou, F. Mohren, A. Gazda, H. Hasenauer, Growing non-native trees in european forests brings benefits and opportunities but also has its risks and limits. *Curr. For. Rep.* **6**, 339–353 (2020).

22. European Forest Institute, *Douglas-Fir—An Option to Europe*, vol. 11 of *What Science Can Tell Us* (European Forest Institute, 2020); https://efi.int/publications-bank/russian-forests-and-climate-change.

23. L. Baeten, H. Bruelheide, F. Van Der Plas, S. Kambach, S. Ratcliffe, T. Jucker, E. Allan, E. Ampoorter, L. Barbaro, C. C. Bastias, J. Bauhus, R. Benavides, D. Bonal, O. Bouriaud, F. Bussotti, M. Carnol, B. Castagneyrol, Y. Charbonnier, E. Chećko, D. A. Coomes, J. Dahlgren, S. M. Dawud, H. De Wandeler, T. Domisch, L. Finér, M. Fischer, M. Fotelli, A. Gessler, C. Grossiord, V. Guyot, S. Hättenschwiler, H. Jactel, B. Jaroszewicz, F. Joly, J. Koricheva, A. Lehtonen, S. Müller, B. Muys, D. Nguyen, M. Pollastrini, K. Radoglou, K. Raulund-Rasmussen, P. Ruiz-Benito, F. Selvi, J. Stenlid, F. Valladares, L. Vesterdal, K. Verheyen, C. Wirth, M. A. Zavala, M. Scherer-Lorenzen, Identifying the tree species compositions that maximize ecosystem functioning in European forests. *J. Appl. Ecol.* **56**, 733–744 (2019).

24. B. Muys, C. Messier, Climate-smart forest management caught between a rock and a hard place. *Ann. For. Sci.* **80**, 43 (2023).

25. J.-Z. Lu, S. Scheu, Response of soil microbial communities to mixed beech-conifer forests varies with site conditions. *Soil Biol. Biochem.* **155**, 108155 (2021).

26. A. Schuldt, P. Huke, J. Glatthorn, J. Hagge, B. Wildermuth, D. Matevski, Tree mixtures mediate negative effects of introduced tree species on bird taxonomic and functional diversity. *J. Appl. Ecol.* **59**, 3049–3060 (2022).

27. T. Wohlgemuth, M. M. Gossner, T. Campagnaro, H. Marchante, M. Van Loo, G. Vacchiano, P. Castro-Díez, D. Dobrowolska, A. Gazda, S. Keren, Z. Keserű, M. Koprowski, N. La Porta, V. Marozas, P. H. Nygaard, V. Podrázský, R. Puchałka, O. Reisman-Berman, L. Straigytė, T. Ylioja, E. Pötzelsberger, J. S. Silva, Impact of non-native tree species in Europe on soil properties and biodiversity: A review. *NeoBiota* **78**, 45–69 (2022).

28. D. M. Richardson, Forestry trees as invasive aliens. *Conserv. Biol.* **12**, 18–26 (1998).

29. D. Liu, F. Essl, B. Lenzner, D. Moser, P. Semenchuk, T. M. Blackburn, P. Cassey, D. Biancolini, C. Capinha, W. Dawson, E. E. Dyer, B. Guénard, E. P. Economo, H. Kreft, J. Pergl, P. Pyšek, M. van Kleunen, C. Rondinini, H. Seebens, P. Weigelt, M. Winter, A. Purvis, S. Dullinger, Regional invasion history and land use shape the prevalence of non-native species in local assemblages. *Glob. Change Biol.* **30**, e17426 (2024).

30. M. Belluau, A. Paquette, D. Gravel, P. B. Reich, A. Stefanski, C. Messier, Exotics are more complementary over time in tree biodiversity–ecosystem functioning experiments. *Funct. Ecol.* **35**, 2550–2561 (2021).

31. S. Ratcliffe, C. Wirth, T. Jucker, F. Van Der Plas, M. Scherer-Lorenzen, K. Verheyen, E. Allan, R. Benavides, H. Bruelheide, B. Ohse, A. Paquette, E. Ampoorter, C. C. Bastias, J. Bauhus, D. Bonal, O. Bouriaud, F. Bussotti, M. Carnol, B. Castagneyrol, E. Chećko, S. M. Dawud, H. D. Wandeler, T. Domisch, L. Finér, M. Fischer, M. Fotelli, A. Gessler, A. Granier, C. Grossiord, V. Guyot, J. Haase, S. Hättenschwiler, H. Jactel, B. Jaroszewicz, F. Joly, S. Kambach, S. Kolb, J. Koricheva, M. Liebersgesell, H. Milligan, S. Müller, B. Muys, D. Nguyen, C. Nock, M. Pollastrini, O. Purschke, K. Radoglou, K. Raulund-Rasmussen, F. Roger, P. Ruiz-Benito, R. Seidl, F. Selvi, I. Seiferling, J. Stenlid, F. Valladares, L. Vesterdal, L. Baeten, Biodiversity and ecosystem functioning relations in European forests depend on environmental context. *Ecol. Lett.* **20**, 1414–1426 (2017).

32. L. Gamfeldt, T. Snäll, R. Bagchi, M. Jonsson, L. Gustafsson, P. Kjellander, M. C. Ruiz-Jaen, M. Fröberg, J. Stendahl, C. D. Philipson, G. Mikusiński, E. Andersson, B. Westerlund, H. Andrén, F. Moberg, J. Moen, J. Bengtsson, Higher levels of multiple ecosystem services are found in forests with more tree species. *Nat. Commun.* **4**, 1340 (2013).

33. S. Soliveres, F. van der Plas, P. Manning, D. Prati, M. M. Gossner, S. C. Renner, F. Alt, H. Arndt, V. Baumgartner, J. Binkenstein, K. Birkhofer, S. Blaser, N. Blüthgen, S. Boch, S. Böhm, C. Börschig, F. Buscot, T. Diekötter, J. Heinze, N. Hölzel, K. Jung, V. H. Klaus, T. Kleinebecker, S. Klemmer, J. Krauss, M. Lange, E. K. Morris, J. Müller, Y. Oelmann, J. Overmann, E. Pašalić, M. C. Rillig, H. M. Schaefer, M. Schloter, B. Schmitt, I. Schöning, M.

Schrumpf, J. Sikorski, S. A. Socher, E. F. Solly, I. Sonnemann, E. Sorkau, J. Steckel, I. Steffan-Dewenter, B. Stempfhuber, M. Tschapka, M. Türke, P. C. Venter, C. N. Weiner, W. W. Weisser, M. Werner, C. Westphal, W. Wilcke, V. Wolters, T. Wubet, S. Wurst, M. Fischer, E. Allan, Biodiversity at multiple trophic levels is needed for ecosystem multifunctionality. *Nature* **536**, 456–459 (2016).

34. A. Hector, R. Bagchi, Biodiversity and ecosystem multifunctionality. *Nature* **448**, 188–190 (2007).

35. E. S. Zavaleta, J. R. Pasari, K. B. Hulvey, G. D. Tilman, Sustaining multiple ecosystem functions in grassland communities requires higher biodiversity. *Proc. Natl. Acad. Sci. U.S.A.* **107**, 1443–1446 (2010).

36. A. Wurz, T. Tscharntke, D. A. Martin, K. Osen, A. A. N. A. Rakotomalala, E. Raveloaritiana, F. Andrianisaina, S. Dröge, T. R. Fulgence, M. R. Soazafy, R. Andriafanomezantsoa, A. Andrianarimisa, F. S. Babarezoto, J. Barkmann, H. Hänke, D. Hölscher, H. Kreft, B. Rakouth, N. R. Guerrero-Ramírez, H. L. T. Ranarijaona, R. Randriamanantena, F. M. Ratsoavina, L. H. Raveloson Ravaomanarivo, I. Grass, Win-win opportunities combining high yields with high multi-taxa biodiversity in tropical agroforestry. *Nat. Commun.* **13**, 4127 (2022).

37. I. Grass, C. Kubitza, V. V. Krishna, M. D. Corre, O. Mußhoff, P. Pütz, J. Drescher, K. Rembold, E. S. Ariyanti, A. D. Barnes, N. Brinkmann, U. Brose, B. Brümmer, D. Buchori, R. Daniel, K. F. A. Darras, H. Faust, L. Fehrmann, J. Hein, N. Hennings, P. Hidayat, D. Hölscher, M. Jochum, A. Knohl, M. M. Kotowska, V. Krashevska, H. Kreft, C. Leuschner, N. J. S. Lobite, R. Panjaitan, A. Polle, A. M. Potapov, E. Purnama, M. Qaim, A. Röll, S. Scheu, D. Schneider, A. Tjoa, T. Tscharntke, E. Veldkamp, M. Wollni, Trade-offs between multifunctionality and profit in tropical smallholder landscapes. *Nat. Commun.* **11**, 1186 (2020).

38. C. Paul, T. Knoke, Forest value: More than commercial. *Science* **354**, 1541 (2016).

39. C. Paul, N. Hanley, S. T. Meyer, C. Fürst, W. W. Weisser, T. Knoke, On the functional relationship between biodiversity and economic value. *Sci. Adv.* **6**, eaax7712 (2020).

40. C. Paul, S. Brandl, S. Friedrich, W. Falk, F. Härtl, T. Knoke, Climate change and mixed forests: How do altered survival probabilities impact economically desirable species proportions of Norway spruce and European beech? *Ann. For. Sci.* **76**, 14 (2019).

41. T. Knoke, E. Gosling, D. Thom, C. Chreptun, A. Rammig, R. Seidl, Economic losses from natural disturbances in Norway spruce forests—A quantification using Monte-Carlo simulations. *Ecol. Econ.* **185**, 107046 (2021).

42. T. Knoke, M. Kindu, I. Jarisch, E. Gosling, S. Friedrich, K. Bödeker, C. Paul, How considering multiple criteria, uncertainty scenarios and biological interactions may influence the optimal silvicultural strategy for a mixed forest. *Forest Policy Econ.* **118**, 102239 (2020).

43. G. von Arnim, B. Möhring, C. Paul, Constrained liquidity during forest calamities: An explorative study for adaptation in private forest enterprises in Germany. *Austrian J. For. Sci.* **138**, 395–412 (2021).

44. P. Feil, C. Neitzel, B. Seintsch, M. Dieter, Privatwaldeigentümer in Deutschland: Ergebnisse einer bundesweiten Telefonbefragung von Personen mit und ohne Waldeigentum. *Landbauforsch Appl Agric Res* **68**, 87–130 (2018).

45. K. F. Wiersum, B. H. M. Elands, M. A. Hoogstra, Small-scale forest ownership across Europe: Characteristics and future potential. *Small-Scale For. Econ. Manag. Policy* **4**, 1–19 (2005).

46. M. Neyret, S. Peter, G. Le Provost, S. Boch, A. L. Boesing, J. M. Bullock, N. Hölzel, V. H. Klaus, T. Kleinebecker, J. Krauss, J. Müller, S. Müller, C. Ammer, F. Buscot, M. Ehbrecht, M. Fischer, K. Goldmann, K. Jung, M. Mehring, T. Müller, S. C. Renner, P. Schall, M. Scherer-Lorenzen, C. Westphal, T. Wubet, P. Manning, Landscape management strategies for multifunctionality and social equity. *Nat. Sustain.* **6**, 391–403 (2023).

47. D. Haase, N. Schwarz, M. Strohbach, F. Kroll, R. Seppelt, Synergies, trade-offs, and losses of ecosystem services in urban regions: An integrated multiscale framework applied to the Leipzig-Halle Region, Germany. *Ecol. Soc.* **17**, art22 (2012).

48. A. F. Cord, B. Bartkowski, M. Beckmann, A. Dittrich, K. Hermans-Neumann, A. Kaim, N. Lienhoop, K. Locher-Krause, J. Priess, C. Schröter-Schlaack, N. Schwarz, R. Seppelt, M. Strauch, T. Václavík, M. Volk, Towards systematic analyses of ecosystem service trade-offs and synergies: Main concepts, methods and the road ahead. *Ecosyst. Serv.* **28**, 264–272 (2017).

49. F. Van Der Plas, S. Ratcliffe, P. Ruiz-Benito, M. Scherer-Lorenzen, K. Verheyen, C. Wirth, M. A. Zavala, E. Ampoorter, L. Baeten, L. Barbaro, C. C. Bastias, J. Bauhus, R. Benavides, A. Benneter, D. Bonal, O. Bouriaud, H. Bruelheide, F. Bussotti, M. Carnol, B. Castagneyrol, Y. Charbonnier, J. H. C. Cornelissen, J. Dahlgren, E. Checko, A. Coppi, S. M. Dawud, M. Deconchat, P. De Smedt, H. De Wandeler, T. Domisch, L. Finér, M. Fotelli, A. Gessler, A. Granier, C. Grossiord, V. Guyot, J. Haase, S. Hättenschwiler, H. Jactel, B. Jaroszewicz, F. Joly, T. Jucker, S. Kambach, G. Kaendler, J. Kattge, J. Koricheva, G. Kunstler, A. Lehtonen, M. Liebergesell, P. Manning, H. Milligan, S. Müller, B. Muys, D. Nguyen, C. Nock, B. Ohse, A. Paquette, J. Peñuelas, M. Pollastrini, K. Radoglou, K. Raulund-Rasmussen, F. Roger, R. Seidl, F. Selvi, J. Stenlid, F. Valladares, J. Van Keer, L. Vesterdal, M. Fischer, L. Gamfeldt, E. Allan, Continental mapping of forest ecosystem functions reveals a high but unrealised potential for forest multifunctionality. *Ecol. Lett.* **21**, 31–42 (2018).

50. C. Leuschner, H. Ellenberg, *Ecology of Central European Forests* (Springer Cham, 2017).

51. BMEL—Federal Ministry of Food and Agriculture, The Forests in Germany—Selected Results of the Third National Forest Inventory (2015).

52. L. Bouriaud, L. Nichiforel, G. Weiss, A. Bajraktari, M. Curovic, Z. Dobsinska, P. Glavonjic, V. Jarský, Z. Sarvasova, M. Teder, Z. Zalite, Governance of private forests in Eastern and Central Europe: An analysis of forest harvesting and management rights. *Ann. For. Res.* **56**, 199–215 (2013).

53. H. Spiecker, Silvicultural management in maintaining biodiversity and resistance of forests in Europe—Temperate zone. *J. Environ. Manage.* **67**, 55–65 (2003).

54. G. Caudullo, W. Tinner, D. de Rigo, *Picea abies* in Europe: Distribution, habitat, usage and threats, in *European Atlas of Forest Tree Species* (European Commission, Luxembourg: Publication Office of the European Union, 2016), pp. 114–116.

55. V. Vitali, U. Büntgen, J. Bauhus, Silver fir and Douglas fir are more tolerant to extreme droughts than Norway spruce in south-western Germany. *Glob. Change Biol.* **23**, 5108–5119 (2017).

56. J. M. Fuchs, A. Hittenbeck, S. Brandl, M. Schmidt, C. Paul, Adaptation strategies for spruce forests—Economic potential of bark beetle management and Douglas fir cultivation in future tree species portfolios. *For. Int. J. For. Res.* **95**, 229–246 (2022).

57. M. Schmid, M. Pautasso, O. Holdenrieder, Ecological consequences of Douglas fir (*Pseudotsuga menziesii*) cultivation in Europe. *Eur. J. For. Res.* **133**, 13–29 (2014).

58. J. Leidinger, M. Blaschke, M. Ehrhardt, A. Fischer, M. M. Gossner, K. Jung, S. Kienlein, J. Kózak, B. Michler, R. Mosandl, S. Seibold, K. Wehner, W. W. Weisser, Shifting tree species composition affects biodiversity of multiple taxa in Central European forests. *For. Ecol. Manage.* **498**, 119552 (2021).

59. V. C. Griess, T. Knoke, Bioeconomic modeling of mixed Norway spruce—European beech stands: Economic consequences of considering ecological effects. *Eur. J. For. Res.* **132**, 511–522 (2013).

60. S. Brandl, C. Paul, T. Knoke, W. Falk, The influence of climate and management on survival probability for Germany's most important tree species. *For. Ecol. Manage.* **458**, 117652 (2020).

61. A. Chao, C. Chiu, K. Hu, F. Van Der Plas, M. W. Cadotte, O. Mitesser, S. Thorn, A. S. Mori, M. Scherer-Lorenzen, N. Eisenhauer, C. Bässler, B. M. Delory, H. Feldhaar, A. Fichtner, T. Hothorn, M. K. Peters, K. Pierick, G. Von Oheimb, J. Müller, Hill–Chao numbers allow decomposing gamma multifunctionality into alpha and beta components. *Ecol. Lett.* **27**, e14336 (2024).

62. J.-Z. Lu, C. Bluhm, E. Foltran, C. A. R. Pérez, C. Ammer, T. Caruso, J. Glatthorn, N. Lamersdorf, A. Polle, D. Sandmann, I. Schaefer, A. Schuldt, M. Maraun, S. Scheu, Functional traits in soil-living oribatid mites unveil trophic reorganization in belowground communities by introduced tree species. *Geoderma* **448**, 116947 (2024).

63. L. E. Likulunga, C. A. Rivera Pérez, D. Schneider, R. Daniel, A. Polle, Tree species composition and soil properties in pure and mixed beech-conifer stands drive soil fungal communities. *For. Ecol. Manage.* **502**, 119709 (2021).

64. B. Wildermuth, C. L. Seifert, M. Husemann, A. Schuldt, Metabarcoding reveals that mixed forests mitigate negative effects of non-native trees on canopy arthropod diversity. *Ecol. Appl.* **33**, e2921 (2023).

65. B. Wildermuth, C. Dönges, D. Matevski, A. Penanhoat, C. L. Seifert, D. Seidel, S. Scheu, A. Schuldt, Tree species identity, canopy structure and prey availability differentially affect canopy spider diversity and trophic composition. *Oecologia* **203**, 37–51 (2023).

66. B. Wildermuth, J. Hagge, C. L. Seifert, R. Tjaden, A. Schuldt, Beneficial effects of native broadleaved forests on canopy beetle diversity are not reduced by admixture of non-native conifers. *J. Appl. Ecol.* **61**, 1000–1014 (2024).

67. F. Tinya, B. Kovács, A. Bidló, B. Dima, I. Király, G. Kutszegi, F. Lakatos, Z. Mag, S. Márialigeti, J. Nascimbene, F. Samu, I. Siller, G. Szél, P. Ódor, Environmental drivers of forest biodiversity in temperate mixed forests—A multi-taxon approach. *Sci. Total Environ.* **795**, 148720 (2021).

68. L. Heidrich, S. Bae, S. Levick, S. Seibold, W. Weisser, P. Krzystek, P. Magdon, T. Nauss, P. Schall, A. Serebryanyk, S. Wöllauer, C. Ammer, C. Bässler, I. Doerfler, M. Fischer, M. M. Gossner, M. Heurich, T. Hothorn, K. Jung, H. Kreft, E.-D. Schulze, N. Simons, S. Thorn, J. Müller, Heterogeneity–diversity relationships differ between and within trophic levels in temperate forests. *Nat. Ecol. Evol.* **4**, 1204–1212 (2020).

69. B. Wildermuth, A. Penanhoat, H. Sennhenn-Reulen, D. Matevski, J. Drescher, M. Aubry-Kientz, D. Seidel, A. Schuldt, Canopy structure influences arthropod communities within and beyond tree identity effects: Insights from combining LiDAR data, insecticidal fogging and machine learning regression modelling. *Ecol. Indic.* **160**, 111901 (2024).

70. G. Alberti, C. Nock, F. Fornasier, M. Scherer-Lorenzen, M. De Nobili, A. Peressotti, L. Hoenig, H. Bruelheide, J. Bauhus, Tree functional diversity influences belowground ecosystem functioning. *Appl. Soil Ecol.* **120**, 160–168 (2017).

71. K. Nadrowski, C. Wirth, M. Scherer-Lorenzen, Is forest diversity driving ecosystem function and service? *Curr. Opin. Environ. Sustain.* **2**, 75–79 (2010).

72. A. Paquette, C. Messier, The effect of biodiversity on tree productivity: From temperate to boreal forests. *Glob. Ecol. Biogeogr.* **20**, 170–180 (2011).

73. T. Sun, L. Dong, Y. Zhang, S. Hättenschwiler, W. H. Schlesinger, J. Zhu, B. Berg, E. C. Adair, Y. Fang, S. E. Hobbie, General reversal of N-decomposition relationship during long-term decomposition in boreal and temperate forests. *Proc. Natl. Acad. Sci. U.S.A.* **121**, e2401398121 (2024).

74. S. Fleck, B. Ahrends, J. Sutmöller, M. Albert, J. Evers, H. Meesenburg, Is biomass accumulation in forests an option to prevent climate change induced increases in nitrate concentrations in the North German Lowland? *Forests* **8**, 219 (2017).

75. K. Mrak, E. Covre-Foltran, N. Lamersdorf, Elevated nitrate concentrations in soil solution under pure Douglas fir stands can be lowered by mixing with European beech and by site selection. *For. Ecol. Manage.* **564**, 122004 (2024).

76. J. Zhang, J. Zhao, R. Cheng, Z. Ge, Z. Zhang, Effects of neighborhood competition and stand structure on the productivity of pure and mixed *Larix principis-rupprechtii* forests. *Forests* **13**, 1318 (2022).

77. T. Knoke, B. Stimm, C. Ammer, M. Moog, Mixed forests reconsidered: A forest economics contribution on an ecological concept. *For. Ecol. Manage.* **213**, 102–116 (2005).

78. H. Spellmann, P. Brang, S. Hein, M. Geb, Große Küstentanne, in *Potenziale Und Risiken Eingeführter Baumarten. Baumartenportraits Mit Naturschutzfachlicher Bewertung* (Göttinger Forstwissenschaften, 2015), pp. 29–46.

79. R.-V. Nagel, Roteiche, in *Potenziale Und Risiken Eingeführter Baumarten. Baumartenportraits Mit Naturschutzfachlicher Bewertung* (Göttinger Forstwissenschaften, 2015), pp. 219–267.

80. T. C. Skurski, B. D. Maxwell, L. J. Rew, Ecological tradeoffs in non-native plant management. *Biol. Conserv.* **159**, 292–302 (2013).

81. A. Mölder, P. Meyer, R.-V. Nagel, Integrative management to sustain biodiversity and ecological continuity in Central European temperate oak (*Quercus robur, Q. petraea*) forests: An overview. *For. Ecol. Manage.* **437**, 324–339 (2019).

82. M. Brändle, R. Brandl, Species richness of insects and mites on trees: Expanding Southwood. *J. Anim. Ecol.* **70**, 491–504 (2001).

83. J. M. Fuchs, K. Husmann, J. Schick, M. Albert, J. Lintunen, C. Paul, Severe and frequent extreme weather events undermine economic adaptation gains of tree-species diversification. *Sci. Rep.* **14**, 2140 (2024).

84. J. Bauhus, B. Pokorny, P. J. van der Meer, P. J. Kanowski, M. Kanninen, Ecosystem goods and services—The key for sustainable plantations, in *Ecosystem Goods and Services from Plantation Forests* (Earthscan, 2010).

85. S. Hättenschwiler, A. V. Tiunov, S. Scheu, Biodiversity and litter decomposition in terrestrial ecosystems. *Annu. Rev. Ecol. Evol. Syst.* **36**, 191–218 (2005).

86. M. Delgado-Baquerizo, P. B. Reich, C. Trivedi, D. J. Eldridge, S. Abades, F. D. Alfaro, F. Bastida, A. A. Berhe, N. A. Cutler, A. Gallardo, L. García-Velázquez, S. C. Hart, P. E. Hayes, J.-Z. He, Z.-Y. Hseu, H.-W. Hu, M. Kirchmair, S. Neuhauser, C. A. Pérez, S. C. Reed, F. Santos, B. W. Sullivan, P. Trivedi, J.-T. Wang, L. Weber-Grullon, M. A. Williams, B. K. Singh, Multiple elements of soil biodiversity drive ecosystem functions across biomes. *Nat. Ecol. Evol.* **4**, 210–220 (2020).

87. N. Eisenhauer, R. Ochoa-Hueso, Y. Huang, K. E. Barry, A. Gebler, C. A. Guerra, J. Hines, M. Jochum, K. Andraczek, S. F. Bucher, F. Buscot, M. Ciobanu, H. Chen, R. Junker, M. Lange, A. Lehmann, M. Rillig, C. Römermann, J. Ulrich, A. Weigelt, A. Schmidt, M. Türke, Ecosystem consequences of invertebrate decline. *Curr. Biol.* **33**, 4538–4547.e5 (2023).

88. S. Wagner, C. Collet, P. Madsen, T. Nakashizuka, R. D. Nyland, K. Sagheb-Talebi, Beech regeneration research: From ecological to silvicultural aspects. *For. Ecol. Manage.* **259**, 2172–2182 (2010).

89. J. Fuchs, K. Husmann, H. Bodelschwingh, R. Koster, K. Staupendahl, A. Offer, B. Möhring, C. Paul, woodValuationDE: A consistent framework for calculating stumpage values in Germany (technical note). *Allg. Forst Jagdztg.* **193**, 16–29 (2023).

90. S. Díaz, U. Pascual, M. Stenseke, B. Martín-López, R. T. Watson, Z. Molnár, R. Hill, K. M. A. Chan, I. A. Baste, K. A. Brauman, S. Polasky, A. Church, M. Lonsdale, A. Larigauderie, P. W. Leadley, A. P. E. van Oudenhoven, F. van der Plaat, M. Schröter, S. Lavorel, Y. Aumeeruddy-Thomas, E. Bukvareva, K. Davies, S. Demissew, G. Erpul, P. Failler, C. A. Guerra, C. L. Hewitt, H. Keune, S. Lindley, Y. Shirayama, Assessing nature's contributions to people. *Science* **359**, 270–272 (2018).

91. N. Hanley, C. Perrings, The economic value of biodiversity. *Annu. Rev. Resour. Econ.* **11**, 355–375 (2019).

92. P. Elsasser, K. Alternbrunn, M. Köthke, M. Lorenz, J. Meyerhoff, Spatial distribution of forest ecosystem service benefits in Germany: A multiple benefit-transfer model. *Forests* **12**, 169 (2021).

93. C. Chreptun, A. Ficko, E. Gosling, T. Knoke, Optimizing forest landscape composition for multiple ecosystem services based on uncertain stakeholder preferences. *Sci. Total Environ.* **857**, 159393 (2023).

94. J. Glatthorn, S. Appleby, N. Balkenhol, P. Kriegel, L. E. Likulunga, J. Lu, D. Matevski, A. Polle, H. Riebl, C. A. Rivera Pérez, S. Scheu, A. Seinsche, P. Schall, A. Schuldt, S. Wingender,

C. Ammer, Species diversity of forest floor biota in non-native Douglas-fir stands is similar to that of native stands. *Ecosphere* **14**, e4609 (2023).

95. S. Heinrichs, C. Ammer, M. Mund, S. Boch, S. Budde, M. Fischer, J. Müller, I. Schöning, E.-D. Schulze, W. Schmidt, M. Weckesser, P. Schall, Landscape-scale mixtures of tree species are more effective than stand-scale mixtures for biodiversity of vascular plants bryophytes and lichens. *Forests* **10**, 73 (2019).

96. P. Schall, S. Heinrichs, C. Ammer, M. Ayasse, S. Boch, F. Buscot, M. Fischer, K. Goldmann, J. Overmann, E.-D. Schulze, J. Sikorski, W. W. Weisser, T. Wubet, M. M. Gossner, Can multi-taxa diversity in European beech forest landscapes be increased by combining different management systems? *J. Appl. Ecol.* **57**, 1363–1375 (2020).

97. P. Schall, M. M. Gossner, S. Heinrichs, M. Fischer, S. Boch, D. Prati, K. Jung, V. Baumgartner, S. Blaser, S. Böhm, F. Buscot, R. Daniel, K. Goldmann, K. Kaiser, T. Kahl, M. Lange, J. Müller, J. Overmann, S. C. Renner, E.-D. Schulze, J. Sikorski, M. Tschapka, M. Türke, W. W. Weisser, B. Wemheuer, T. Wubet, C. Ammer, The impact of even-aged and uneven-aged forest management on regional biodiversity of multiple taxa in European beech forests. *J. Appl. Ecol.* **55**, 267–278 (2018).

98. F. van der Plas, P. Manning, S. Soliveres, E. Allan, M. Scherer-Lorenzen, K. Verheyen, C. Wirth, M. A. Zavala, E. Ampoorter, L. Baeten, L. Barbaro, J. Bauhus, R. Benavides, A. Benneter, D. Bonal, O. Bouriaud, H. Bruelheide, F. Bussotti, M. Carnol, B. Castagneyrol, Y. Charbonnier, D. A. Coomes, A. Coppi, C. C. Bastias, S. M. Dawud, H. De Wandeler, T. Domisch, L. Finér, A. Gessler, A. Granier, C. Grossiord, V. Guyot, S. Hättenschwiler, H. Jactel, B. Jaroszewicz, F. Joly, T. Jucker, J. Koricheva, H. Milligan, S. Mueller, B. Muys, D. Nguyen, M. Pollastrini, S. Ratcliffe, K. Raulund-Rasmussen, F. Selvi, J. Stenlid, F. Valladares, L. Vesterdal, D. Zielínski, M. Fischer, Biotic homogenization can decrease landscape-scale forest multifunctionality. *Proc. Natl. Acad. Sci. U.S.A.* **113**, 3557–3562 (2016).

99. Niedersächsische Landesforsten, LÖWE—Long-term ecological forest development (2024). https://landesforsten.de/wir/loewe/.

100. C. Ammer, P. Annighöfer, N. Balkenhol, D. Hertel, C. Leuschner, A. Polle, N. Lamersdorf, S. Scheu, J. Glatthorn, RTG 2300—Enrichment of European beech forests with conifers. *PANGAEA* https://doi.org/10.1594/PANGAEA.925228 (2020).

101. FAO, *World Reference Base for Soil Resources 2014* (Food and Agriculture Organization of the United Nations, 2015).

102. E. Foltran, C. Ammer, N. Lamersdorf, Do admixed conifers change soil nutrient conditions of European beech stands? *Soil Res.* **61**, 647–662 (2023).

103. C. A. Rivera Pérez, D. Janz, D. Schneider, R. Daniel, A. Polle, Transcriptional landscape of ectomycorrhizal fungi and their host provides insight into N uptake from forest soil. *mSystems* **7**, e0095721 (2022).

104. S. M. Appleby, N. Balkenhol, Douglas fir and Norway spruce have similar effects on small mammal density, but not survival, in Central European managed forests. *Mamm. Biol.* **104**, 25–39 (2024).

105. A. S. Lwila, M. Mund, C. Ammer, J. Glatthorn, Site conditions more than species identity drive fine root biomass, morphology and spatial distribution in temperate pure and mixed forests. *For. Ecol. Manage.* **499**, 119581 (2021).

106. D. I. Forrester, I. H. H. Tachauer, P. Annighoefer, I. Barbeito, H. Pretzsch, R. Ruiz-Peinado, H. Stark, G. Vacchiano, T. Zlatanov, T. Chakraborty, S. Saha, G. W. Sileshi, Generalized biomass and leaf area allometric equations for European tree species incorporating stand structure, tree age and climate. *For. Ecol. Manage.* **396**, 160–175 (2017).

107. T. Beck, R. G. Joergensen, E. Kandeler, F. Makeschin, E. Nuss, H. R. Oberholzer, S. Scheu, An inter-laboratory comparison of ten different ways of measuring soil microbial biomass C. *Soil Biol. Biochem.* **29**, 1023–1032 (1997).

108. J. Hansen, J. Nagel, *Waldwachstumskundliche Softwaresysteme auf Basis von TreeGrOSS—Anwendung und theoretische Grundlagen* (Univ.-Verl. Göttingen, 2014), *Beiträge aus der Nordwestdeutschen Forstlichen Versuchsanstalt*.

109. M. Schmidt, Standortsensitive und kalibrierbare Bonitätsfächer: Wachstumspotenziale wichtiger Baumarten unter Klimawandel. *Allg Forst Jagdztg.* **190**, 136–160 (2020).

110. W. Enke, T. Deutschländer, F. Schneider, W. Küchler, Results of five regional climate studies applying a weather pattern based downscaling method to ECHAM4 climate simulation. *Meteorol. Z.* **14**, 247–257 (2005).

111. G. Martin, N. Bellouin, W. Collins, I. Culverwell, P. Halloran, S. Hardiman, T. Hinton, C. Jones, R. McDonald, A. McLaren, F. O'Connor, M. Roberts, J. Rodriguez, S. Woodward, M. Best, M. Brooks, A. Brown, N. Butchart, C. Dearden, A. Wiltshire, The HadGEM2 family of Met Office Unified Model Climate configurations. *Geosci. Model Dev. Discuss.* **4**, 723–757 (2011).

112. F. Kreienkamp, A. Paxian, B. Früh, P. Lorenz, C. Matulla, Evaluation of the empirical–statistical downscaling method EPISODES. *Climate Dynam.* **52**, 991–1026 (2019).

113. H. Bodelschwingh, "Ökonomische Potentiale von Waldbeständen," thesis, Fakultät für Forstwissenschaften und Waldökologie, University of Göttingen, Göttingen (2018).

114. A. Offer, K. Staupendahl, *Holzwerbungskosten- und Bestandessortentafeln* (HessenForst, 2018).

115. B. Möhring, Nachhaltige Forstwirtschaft und Rentabilitätsrechnung–ein Widerspruch? *Allg. Forst Jagdztg.* **172**, 61–66 (2001).

116. R. J. Hijmans, S. E. Cameron, J. L. Parra, P. G. Jones, A. Jarvis, Very high resolution interpolated climate surfaces for global land areas. *Int. J. Climatol.* **25**, 1965–1978 (2005).

117. J. Oksanen, G. Simpson, F. G. Blanchet, R. Kindt, P. Legendre, P. Minchin, R. O'hara, P. Solymos, H. Stevens, E. Szöcs, H. Wagner, M. Barbour, M. Bedward, B. Bolker, D. Borcard, G. Carvalho, M. Chirico, M. De Cáceres, S. Durand, J. Weedon, Vegan Community Ecology Package Version 2.6–2 April 2022 (2022).

118. C. Gao, A. Dusa, ggVennDiagram: A "ggplot2" Implement of Venn Diagram (2024). https://CRAN.R-project.org/package=ggVennDiagram.

119. T. Wei, V. Simko, R package "corrplot": Visualization of a Correlation Matrix (Version 0.92) (2021). https://github.com/taiyun/corrplot.

120. J. E. K. Byrnes, L. Gamfeldt, F. Isbell, J. S. Lefcheck, J. N. Griffin, A. Hector, B. J. Cardinale, D. U. Hooper, L. E. Dee, J. E. Duffy, Investigating the relationship between biodiversity and ecosystem multifunctionality: Challenges and solutions. *Methods Ecol. Evol.* **5**, 111–124 (2014).

121. M. E. Brooks, K. Kristensen, K. J. van Benthem, A. Magnusson, C. W. Berg, A. Nielsen, H. J. Skaug, M. Mächler, B. M. Bolker, glmmTMB balances speed and flexibility among packages for zero-inflated generalized linear mixed modeling. *R J.* **9**, 378 (2017).

122. D. Lüdecke, Package 'performance' (2024). https://cran.r-project.org/web/packages/performance/performance.pdf.

123. Hartig, Florian, DHARMa: Residual Diagnostics for Hierarchical (Multi-Level/Mixed) Regression Models (2022). https://CRAN.R-project.org/package=DHARMa.

124. T. Hothorn, F. Bretz, P. Westfall, Simultaneous inference in general parametric models. *Biom. J.* **50**, 346–363 (2008).

125. D. Lüdecke, ggeffects: Tidy data frames of marginal effects from regression models. *J. Open Source Softw.* **3**, 772 (2018).

126. M. Loreau, Separating sampling and other effects in biodiversity experiments. *Oikos* **82**, 600 (1998).

127. D. A. Martin, F. Andrianisaina, T. R. Fulgence, K. Osen, A. A. N. A. Rakotomalala, E. Raveloaritiana, M. R. Soazafy, A. Wurz, R. Andriafanomezantsoa, H. Andriamaniraka, A. Andrianarimisa, J. Barkmann, S. Dröge, I. Grass, N. Guerrero-Ramirez, H. Hänke, D. Hölscher, B. Rakouth, H. L. T. Ranarijaona, R. Randriamanantena, F. M. Ratsoavina, L. H. R. Ravaomanarivo, D. Schwab, T. Tscharntke, D. C. Zemp, H. Kreft, Land-use trajectories for

sustainable land system transformations: Identifying leverage points in a global biodiversity hotspot. *Proc. Natl. Acad. Sci. U.S.A.* **119**, e2107747119 (2022).

128. A. Canty, B. Ripley, boot: Bootstrap R (S-Plus) Functions (2024).

129. B. Schloerke, D. Cook, J. Larmarange, GGally: Extension to “ggplot2” (2021). https://CRAN.R-project.org/package=GGally.

130. R Core Team, R: A language and environment for statistical computing, R Foundation for Statistical Computing (2020). https://R-project.org/.

131. D. P. Faith, Valuation and appreciation of biodiversity: The “Maintenance of Options” provided by the variety of life. *Front. Ecol. Evol.* **9**, doi.org/10.3389/fevo.2021.635670 (2021).

132. N. Cools, L. Vesterdal, B. De Vos, E. Vanguelova, K. Hansen, Tree species is the major factor explaining C:N ratios in European forest soils. *For. Ecol. Manage.* **311**, 3–16 (2014).

133. R. Lal, Soil carbon sequestration impacts on global climate change and food security. *Science* **304**, 1623–1627 (2004).

134. K. Paustian, J. Lehmann, S. Ogle, D. Reay, G. P. Robertson, P. Smith, Climate-smart soils. *Nature* **532**, 49–57 (2016).

135. P. Baldrian, Forest microbiome: Diversity, complexity and dynamics. *FEMS Microbiol. Rev.* **4**, 109–130 (2016).

136. M. Wang, J. Cui, H. Liu, X. Xu, Characterization of soil microbial biomass carbon and nitrogen in four forest types of shushan urban forest park. *Forests* **14**, 1498 (2023).

137. A. M. Potapov, A. A. Goncharov, E. E. Semenina, A. Y. Korotkevich, S. M. Tsurikov, O. L. Rozanova, A. E. Anichkin, A. G. Zuev, E. S. Samoylova, I. I. Semenyuk, I. V. Yevdokimov, A. V. Tiunov, Arthropods in the subsoil: Abundance and vertical distribution as related to soil organic matter, microbial biomass and plant roots. *Eur. J. Soil Biol.* **82**, 88–97 (2017).

138. X. Wan, Z. Huang, Z. He, Z. Yu, M. Wang, M. R. Davis, Y. Yang, Soil C:N ratio is the major determinant of soil microbial community structure in subtropical coniferous and broadleaf forest plantations. *Plant and Soil* **387**, 103–116 (2015).

139. M. P. Krishna, M. Mohan, Litter decomposition in forest ecosystems: A review. *Energy Ecol. Environ.* **2**, 236–249 (2017).

140. M. Giweta, Role of litter production and its decomposition, and factors affecting the processes in a tropical forest ecosystem: A review. *J. Ecol. Environ.* **44**, 11 (2020).

141. M. L. McCormack, I. A. Dickie, D. M. Eissenstat, T. J. Fahey, C. W. Fernandez, D. Guo, H.-S. Helmisaari, E. A. Hobbie, C. M. Iversen, R. B. Jackson, J. Leppälammi-Kujansuu, R. J. Norby, R. P. Phillips, K. S. Pregitzer, S. G. Pritchard, B. Rewald, M. Zadworny, Redefining fine roots improves understanding of below-ground contributions to terrestrial biosphere processes. *New Phytol.* **207**, 505–518 (2015).

142. C. Leuschner, D. Hertel, I. Schmid, O. Koch, A. Muhs, D. Hölscher, Stand fine root biomass and fine root morphology in old-growth beech forests as a function of precipitation and soil fertility. *Plant and Soil* **258**, 43–56 (2004).

143. E. Laliberté, Below-ground frontiers in trait-based plant ecology. *New Phytol.* **213**, 1597–1603 (2017).

144. J. M. Chen, T. A. Black, Defining leaf area index for non-flat leaves. *Plant Cell Environ.* **15**, 421–429 (1992).

145. N. J. J. Bréda, Ground-based measurements of leaf area index: A review of methods, instruments and current controversies. *J. Exp. Bot.* **54**, 2403–2417 (2003).

146. F. H. Härtl, S. Höllerl, T. Knoke, A new way of carbon accounting emphasises the crucial role of sustainable timber use for successful carbon mitigation strategies. *Mitig. Adapt. Strateg. Glob. Change* **22**, 1163–1192 (2017).

147. K. I. Paul, K. Jacobsen, V. Koul, P. Leppert, J. Smith, Predicting growth and sequestration of carbon by plantations growing in regions of low-rainfall in southern Australia. *For. Ecol. Manage.* **254**, 205–216 (2008).

148. R. Sedjo, B. Sohngen, Carbon sequestration in forests and soils. *Annu. Rev. Resour. Econ.* **4**, 127–144 (2012).

149. J. Schelhas, T. J. Brandeis, T. K. Rudel, Planted forests and natural regeneration in forest transitions: Patterns and implications from the U.S. *South. Reg. Environ. Change* **21**, 8 (2021).

150. E. van Meijgaard, L. H. van Ulft, W. J. van de Berg, F. C. Bosveld, B. J. J. M. van den Hurk, G. Lenderink, A. P. Siebesma, The KNMI regional atmospheric climate model RACMO, version 2.1. *R. Neth. Meteorol. Inst.* (2008).

151. W. Hazeleger, X. Wang, C. Severijns, S. Ştefănescu, R. Bintanja, A. Sterl, K. Wyser, T. Semmler, S. Yang, B. van den Hurk, T. van Noije, E. van der Linden, K. van der Wiel, EC-Earth V2.2: Description and validation of a new seamless earth system prediction model. *Climate Dynam.* **39**, 2611–2629 (2012).

152. M. Albert, J. Nagel, M. Schmidt, R.-V. Nagel, H. Spellmann, Eine neue Generation von Ertragstafeln für Eiche, Buche, Fichte, Douglasie und Kiefer, version 1.0, Zenodo (2021). https://doi.org/10.5281/zenodo.6343907.
